# The impact of face-mask mandates on all-cause mortality in Switzerland: a quasi-experimental study

**DOI:** 10.1093/eurpub/ckac123

**Published:** 2022-09-10

**Authors:** Giacomo De Giorgi, Pascal Geldsetzer, Felix Michalik, M Maddalena Speziali

**Affiliations:** Institute of Economics and Econometrics, Geneva School of Economics and Management, University of Geneva, Geneva 4, Switzerland; BREAD, Bureau for Research and Economic Analysis of Development, E Providence, RI, USA; CEPR, Centre for Economic Policy Research, London, UK; IPA, Innovations for Poverty Action, Washington, DC, USA; Division of Primary Care and Population Health, Department of Medicine, Stanford University, Stanford, CA, USA; Chan Zuckerberg Biohub, San Francisco, CA, USA; Heidelberg Institute of Global Health, Heidelberg University, Heidelberg, Germany; Institute of Economics and Econometrics, Geneva School of Economics and Management, University of Geneva, Geneva 4, Switzerland; University Magna Graecia of Catanzaro, Catanzaro, Italy

## Abstract

**Background:**

Whereas there is strong evidence that wearing a face mask is effective in reducing the spread of the severe acute respiratory syndrome coronavirus 2 (SARS-CoV-2), evidence on the impact of mandating the wearing of face masks on deaths from coronavirus disease 2019 (COVID-19) and all-cause mortality is more sparse and likely to vary by context. Focusing on a quasi-experimental setting in Switzerland, we aimed to determine (i) the effect of face-mask mandates for indoor public spaces on all-cause mortality; and (ii) how the effect has varied over time, and by age and sex.

**Methods:**

Our analysis exploited the fact that between July and October 2020, nine cantons in Switzerland extended a face-mask mandate at different time points from being restricted to public transportation only to applying to all public indoor places. We used both a Difference-in-Differences approach with fixed-effects for canton and week and an event-study approach.

**Results:**

In our main Difference-in-Differences model, the face-mask mandate was associated with a 0.3% reduction in all-cause mortality [95% confidence interval (CI): −3.4% to 2.7%; *P* = 0.818]. This null effect was confirmed in the event-study approach and a variety of robustness checks. Combining the face-mask mandate with social distancing rules led to an estimated 5.1% (95% CI: −7.9% to −2.4%; *P* = 0.001) reduction in all-cause mortality.

**Conclusions:**

Mandating face-mask use in public indoor spaces in Switzerland in mid-to-late 2020 does not appear to have resulted in large reductions in all-cause mortality in the short term. There is some suggestion that combining face-mask mandates with social distancing rules reduced all-cause mortality.

## Introduction

Since the beginning of the COVID-19 pandemic, governments have resorted to several non-pharmaceutical interventions (NPIs) in their response to COVID-19 including social distancing rules, contact tracing, face-mask mandates and partial or total lockdowns. Despite the rollout of vaccines, NPIs still remain a commonly implemented policy tool for COVID-19 in 2021. However, there is still debate on which combination of NPIs is most effective in preventing overburdening of the health system and reducing morbidity and mortality.

The effectiveness of wearing face masks in reducing the probability of COVID-19 transmission is well established.^1^ However, the effectiveness of face-mask mandates is less certain and likely depends on a host of factors including the degree to which the population adheres to the mandate, which population groups adhere more strictly, the type of mask worn, and the dynamics of the epidemic. A systematic review of NPIs identified one randomized controlled trial (RCT) in addition to 34 observational studies that estimate individual interventions.^2^ The RCT was conducted in Denmark and was underpowered to evaluate the effect of face masks.^3^ Eight studies were included in a meta-analysis, which suggested a relative reduction in the incidence of COVID-19 of 53% [risk ratio: 0.47, 95% confidence interval (CI): 0.29–0.75] for mask wearing. There was, however, high heterogeneity of effect sizes between the studies. The review also mentioned a large-scale cluster-randomized trial of community-level face-mask promotion that was conducted in rural Bangladesh.^4^ With a relative prevalence reduction of 11% (risk ratio: 0.89, 95% CI: 0.78–1.00), this trial found a statistically significant, but much smaller, effect size of face masks. It is unclear to what degree these findings from rural Bangladesh are generalizable to Switzerland.

The empirical evaluations of face-mask mandates have largely focused on their impact on the rate of growth of new cases of the severe acute respiratory syndrome coronavirus 2 (SARS-CoV-2) infection and deaths due to coronavirus disease 2019 (COVID-19).^5–10^ These outcomes are vulnerable to substantial measurement error including from the degree of testing that is being conducted, attribution of deaths to COVID-19 as opposed to underlying health problems, and variation in the quality of, and access to, healthcare. They also fail to measure the indirect effects of face-mask mandates, such as those deaths that are averted because the mandate reduced demand on hospitals, which as a result may have been able to provide better care to non-COVID-19 patients. In our analysis, we focued on all-cause mortality as the primary outcome. Unlike COVID-19-specific outcomes, all-cause mortality in our study setting (Switzerland) is recorded highly reliably. Arguably, it is also the health outcome that is of highest importance to society. Nonetheless, to be comprehensive in our approach, we also studied the effect of the mandates on COVID-19 cases and deaths. We exploited a quasi-experimental setting that enables us to obtain causal effect estimates under weaker assumptions than most existing evaluations. Specifically, our analysis exploited variation in both the timing and geography of face-mask mandates resulting from the fact that public health policies in Switzerland have differed across cantons and have interacted with Federation-wide policies that were instituted at certain time points in the pandemic. Cantons that did not introduce face-mask mandates serve as controls and allow us to compare the actual outcome after mandate introduction with its counterfactual. NPIs might show an effect only after a couple of days past their introduction^11^^,^^12^; we examined this issue further in our analyses.

The aims of this study were 2-fold: to determine (i) the effect of face-mask mandates for indoor public spaces on all-cause mortality; and (ii) how the effect of face-mask mandates on all-cause mortality has varied over time, and by age and sex. In secondary analyses, we examined the effect of the mandates on new COVID-19 cases and deaths due to COVID-19. In addition, we considered the effect of adding contact tracing and stricter social distancing rules to face-mask mandates.

## Methods

### Study setting and policy environment

While initially the Swiss Federal Council centralized COVID-19 measures in the spring of 2020 and mandated face-mask wearing on all Swiss public transportation on 6 July 2020, it returned some autonomy back to the cantons in the summer of 2020. As a result, public health policies differed across cantons. Between 7 July and 3 September 2020, 9 cantons extended face-mask requirements to all public (indoor) places, e.g. supermarkets, stores, and restaurants (which we refer to as *treated*), while the other 17 cantons did not (which serve as our *controls*). This study is concerned with ascertaining the causal effect of extending face-mask requirements to these additional locations by employing Difference-in-Differences type models.

On 18 October 2020, the Federation applied the face-mask requirement to all public indoor spaces in the whole country.

In addition to face-mask mandates, some cantons introduced social distancing rules, which refer in particular to policies limiting the number of guests at restaurants and events, as well as contact tracing policies. Supplementary text S1 and Supplementary figures S1 and S2 provide more details on all policies and their timing.

### Data sources and primary outcome

Our analysis is based on a longitudinal dataset at the canton-week-year level. The dataset comprises observations for the 26 Swiss cantons in the first 40 weeks of each year between 2012 and 2020. We chose 40 weeks (rather than 42 weeks) as the study length because we assumed a minimum of 2 weeks for the first effects of a face-mask mandate to be observable. Five cantons implemented face-mask mandates after these 40 weeks but prior to 18 October 2020 (10 October for Zug and Ticino, 12 October for Bern, and 17 October for Grischun and Luzern).

To construct the policy timeline, we used information from newspaper articles and cantonal official internet web pages. Data on total deaths and population were obtained from the Federal Statistical Office website (FSO—Cause of Death Statistics, 2021).^13^ These data disaggregate deaths by sex and 5-year age groups. We grouped age into four categories (0–29 years, 30–59 years, 60–89 years, and 90+ years). We computed all-cause mortality, our main outcome of interest, as deaths per 100 000 residents (see Supplementary text S2 for details). We used the logarithm of all-cause mortality such that the regression coefficients can be interpreted as an approximation of the percentage change in the outcome with every one-unit change in the explanatory variable. We show descriptive statistics of all-cause mortality by age and sex at the national level and by canton in the Supplementary appendix (Supplementary Tables S1 and S2).

### Secondary outcomes

Our secondary outcomes are new COVID-19 cases per week and new deaths from COVID-19 per week. Data on these outcomes were obtained from the Statistical Office of the Canton of Zurich.^14^ COVID-19 cases and deaths were not disaggregated by sex and age category because this information was not available for all cantons. The Federal Council did not instruct cantons on the definition of COVID-19 cases and deaths.^15^ All cantons apart from Geneva, however, confirmed that preliminary diagnoses were counted as cases. Starting on 9 March 2020, the Federal Council advised testing only for (i) individuals with severe symptoms, and (ii) individuals at high risk of complications or in direct contact with patients or residents of retirement and nursing homes. On 27 April 2020, the Federal Office of Public Health additionally recommended testing individuals with symptoms suggestive of acute respiratory disease, muscle pain or loss of smell or taste.

### Statistical analysis

To identify the causal effect of face-mask mandates on our outcomes, we employed a Difference-in-Differences model with fixed effects and an event study. Below, we describe the analyses for all-cause mortality. The approach employed for our secondary outcomes (COVID-19 cases and COVID-19 deaths) was identical to the one used for all-cause mortality. In our research design, a *Treated* canton has a mandate of mask wearing in public indoor spaces while a *Control* canton has compulsory mask wearing on public transport only. Intuitively, the idea is to compare the difference in average outcomes between treated and controls before and after the treatment. Following Solon *et al*.,^16^ we conducted our analyses using cantonal population size as analytic weights.

#### Difference-in-Differences analysis

We estimated the following equation:

(1) $$\log\left(Y_{\mathrm{cwt}}\right)=\;\alpha_{0}+\;\beta_{0}{\mathrm{Treat}}_{c\;}+\;\beta_{1}{\mathrm{Post}}_{wt}+\beta_{2}{\mathrm{Did}}_{\mathrm{cwt}}+\gamma_{c}+\theta_{w}+\tau_{t}+\varepsilon_{\mathrm{cwt}}$$

with *Treat* being a binary variable indicating whether the canton had adopted the face-mask mandate; and *Post* a binary variable taking a value of 1 in the post-mandate period (and 0 otherwise). *Did* is the interaction between *Treat* and *Post*. Our parameter of interest is $\beta_{2}$. Finally, $\gamma_{c}$, $\theta_{w}$ and $\tau_{t}$ are, respectively, binary variables for canton, week and year. The key identifying assumption of Difference-in-Differences analyses is that of common trends between treated and control cantons in the absence of the treatment. Supplementary figures S9–S14 indicate that this assumption is plausible given that treated and control cantons have parallel trends prior to implementation of the face-mask mandate.

Importantly, we set a unique starting date for the post-mandate period for all cantons on July 7. This allows us to eliminate likely anticipation bias due to behavioural responses. For example, people in cantons that implemented the policy at a later date may have decided to wear a face mask in indoor places prior to the canton’s face-mask mandate. However, attributing the treatment earlier than the actual date of adoption in some cantons might lead to a downward estimated coefficient. In practice, to an extreme, this would consider as treated some cantons that would be controls at that point. Thus, we tested whether this attenuation is relevant in the event-study analysis, and in a staggered Difference-in-Differences analysis, which uses the actual date of policy implementation (see Supplementary Tables S11–S15 and text S6).

#### Event-study analysis

As a complementary approach to the Difference-in-Differences analysis described above, we also implemented a panel-event study, with the date of the ‘event’ being the date of implementation of the face-mask mandate in a particular canton. Formally, we estimated the following equation:

(2) $$\log\left(Y_{cw}\right)=\;\alpha_{1}+\sum_{j=2}^{J}\delta_{j}{\left(\mathrm{Lag}\;j\;\right)}_{cw}+\sum_{k=1}^{K}\lambda_{k}{\left(\mathrm{Lead}\;k\;\right)}_{cw}+\mu_{w}+\psi_{c}+\varepsilon_{cw}$$

where *ψ_c_* and μ*_w_* are binary variables for canton and week, and *ε_cw_* is an unobserved error term. Further, *Lag_j_* and *Lead_k_* are two binary variables indicating the number of weeks until implementation of the face-mask mandate in canton *c*. Formally, we defined *Lag_j_* and *Lead_k_* according to Equations (3)–(6):

(3) $${\left(\mathrm{Lag}\;j\;\right)}_{cw}=1[t\leq {\mathrm{Event}}_{c}-J]\;,$$

 

(4) $${\left(\mathrm{Lag}\;j\;\right)}_{cw}=1[t={\mathrm{Event}}_{c}-j]\;\mathrm{for}\;j\in \{1,...,J-1\}\;,$$

 

(5) $${\left(\mathrm{Lead}\;k\;\right)}_{cw}=1[t={\mathrm{Event}}_{c}+k]\;\mathrm{for}\;k\in \{1,...,K-1\}\;,$$

 

(6) $${\left(\mathrm{Lead}\;k\;\right)}_{cw}=1[t\geq {\mathrm{Event}}_{c}+K].$$

where, *Event_c_* is a variable indicating the week *w* in which the face-mask mandate was implemented in canton *c*. The first *Lag* was omitted to capture the baseline difference between treated and control cantons.

#### Effect heterogeneity over time

To assess how the effects of the face-mask mandate vary with time, we estimated a dynamic model where *β* can vary across weeks:

(7) $$\begin{array}{c} \;\text{log}\;\left(Y_{\mathrm{cwt}}\right)=\;\alpha_{2}+\;\beta_{0}{\mathrm{Treat}}_{c\;}+\sum_{w=27\;}^{40}\beta_{1w}\;[{\mathrm{week}}_{cw(t=2020)}-{\mathrm{week}(T=1)}_{c}]\\ +\sum_{w=27\;}^{40}\beta_{2w}\;{\mathrm{Treat}}_{c}[{\mathrm{week}}_{cw(t=2020)}-{\mathrm{week}\;(T=1)}_{c}]\;+\gamma_{c}+\theta_{w}+\tau_{t}+\varepsilon_{\mathrm{cwt}} \end{array}$$

*Treat_c_* is a binary variable equal to 1 if canton *c* is ever treated (i.e. implements a face-mask mandate). Then, [*week* − *week* (*T *=* *1)_*c*_] is the difference between the observation week and the first week of implementation of the extra measure in canton *c*. The parameters of interest are the *β*_2__*w*_, which represent the mean difference in the outcome of interest in a specific week *w*. We also control for canton ($\gamma_{c}$), week ($\theta_{w}$) and year ($\tau_{t}$) fixed-effects.

#### Effect heterogeneity by age group and sex

We first estimated our Difference-in-Differences model (Equation (1)) in each age group, controlling for time and canton fixed-effects. Then, we employed a pooled regression, which allows us to explore the contribution of each age group to the aggregate estimate. In particular, we used the youngest age group (0–29) as baseline and estimated the partial effect for the three other age groups.

### Ethics

This study received a determination of not-human subjects research by the institutional review board of the Universitätsklinikum Heidelberg.

## Results

### Effect on all-cause mortality using a Difference-in-Differences analysis

None of our regression model specifications found a significant effect of the face-mask mandate on all-cause mortality (table 1). The point estimate was −0.003 in all regression model specifications, with the 95% CI ranging from −0.034 to 0.027. Interpreting the regression coefficient as an approximation of the percentage change in the outcome, the point estimate, thus, corresponds to a 0.3% decrease in all-cause mortality, with the 95% CI ranging from −3.4% to 2.7%. The effects remained non-significant when examining all-cause mortality separately by sex [further confirmed by a pooled regression on sex (Supplementary table S10)]. These effect estimates were similar when assigning the same weight to each canton instead of weighting by a canton’s population size (Supplementary table S23).

**Table 1:** Results of the Difference-in-Differences regressions

	(1)	(2)	(3)	(4)	(5)	(6)	(7)	(8)	(9)
Variables	Male	Male	Male	Female	Female	Female	Total	Total	Total
Treat	−0.070	−0.009***	−0.009***	−0.055	0.066***	0.066***	−0.063	0.026***	0.026***
	(0.049)	(0.001)	(0.001)	(0.055)	(0.001)	(0.001)	(0.051)	(0.001)	(0.001)
Post	−0.046**	−0.045**	0.010	−0.116***	−0.115***	−0.024	−0.082***	−0.081***	−0.007
	(0.018)	(0.018)	(0.026)	(0.013)	(0.014)	(0.018)	(0.013)	(0.013)	(0.020)
DiD	−0.031	−0.031	−0.031	0.024	0.024	0.023	−0.003	−0.003	−0.003
	(0.020)	(0.020)	(0.021)	(0.021)	(0.021)	(0.021)	(0.015)	(0.015)	(0.015)
Constant	2.705***	2.617***	2.697***	2.741***	2.622***	2.778***	2.733***	2.625***	2.744***
	(0.041)	(0.001)	(0.014)	(0.045)	(0.001)	(0.017)	(0.042)	(0.001)	(0.012)
Observations	9168	9168	9168	9178	9178	9178	9329	9329	9329
*R*-squared	0.021	0.190	0.286	0.016	0.225	0.371	0.026	0.291	0.462
Year FE	No	No	Yes	No	No	Yes	No	No	Yes
Canton FE	No	Yes	Yes	No	Yes	Yes	No	Yes	Yes
Week FE	No	No	Yes	No	No	Yes	No	No	Yes
Mean	2.686	2.686	2.686	2.719	2.719	2.719	2.708	2.708	2.708

*Note*: Results of regression based on Equation (1); weighted using population as analytical weights. Column 1-2-3 contain observations for male population. Columns 4-5-6 contain observations for the female population. Column 7-8-9 contain observations for aggregate male and female population. S.E. clustered at a canton level (***P < 0.01, **P < 0.05, *P < 0.1). Period of estimation: between January 2012 and 4 October 2020. *Treated* cantons are those that between July 7 and October 4 have imposed any mask requirement other than Federal indications (e.g. in supermarkets, restaurants, open space): BS, FR, GE, JU, NE, SO, VS, VD, ZH. *Post* is equal to 1 for all cantons after July 7.

### Effect on all-cause mortality using an event-study analysis

**Figure 1:**
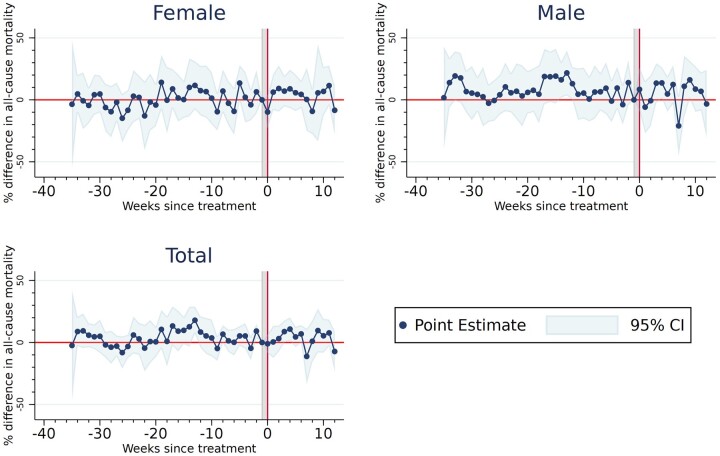
Effect of the face-mask mandate on all-cause mortality using an event-study approach. *Note*: Estimates of Equation (2), weighted using population as analytical weights. Point estimates are displayed along with their 95% confidence intervals. The percentage difference in all-cause mortality is approximated by the log. Baseline period for the analysis: 1 week prior to implementation of the face-mask mandate in each canton, indicated by the vertical line in the plot.

In line with the findings of the Difference-in-Differences analysis, the event-study analysis found no significant effects of the face-mask mandate on all-cause mortality (figure 1), neither for the whole population nor when examining men and women separately. There was no indication that the effect of the face-mask mandate varied depending on the number of weeks since it had been implemented.

### Effect heterogeneity over time

**Figure 2:**
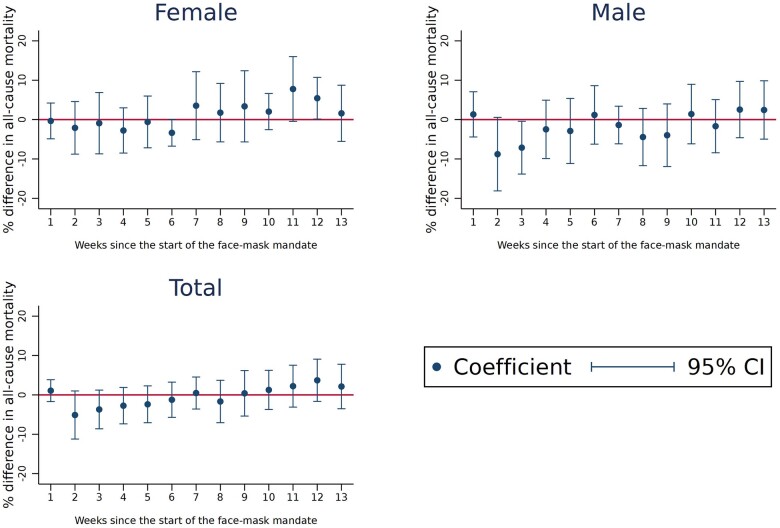
Variation in the effect of the face-mask mandate by time since implementation *Note*: The dots indicate the estimated *β*_2_ in week *w* as in Equation (7), weighted using population as analytical weights. The percentage difference in all-cause mortality is approximated by the log. Week 1 is the first week after the treatment, until the 13th week. Outcome defined as Supplementary equation (S1). Each vertical bar represents the respective 95% confidence interval.

Similar to the event-study approach, our analysis using a Difference-in-Differences approach with a dynamic beta-coefficient found no clear patterns of variation in the effect of the face-mask mandate by the number of weeks since the mandate’s implementation (figure 2).

### Effect heterogeneity by age

None of our analytical approaches—neither the Difference-in-Differences design, event-study approach, nor the Difference-in-Differences approach with dynamic beta-coefficients—found any evidence of variation in the effect of the face-mask mandate by age group (Supplementary appendix tables S3–S9, S12–S15, S17–S20, appendix figures S3–S8).

### Impact on COVID-19 cases and deaths

**Figure 3:**
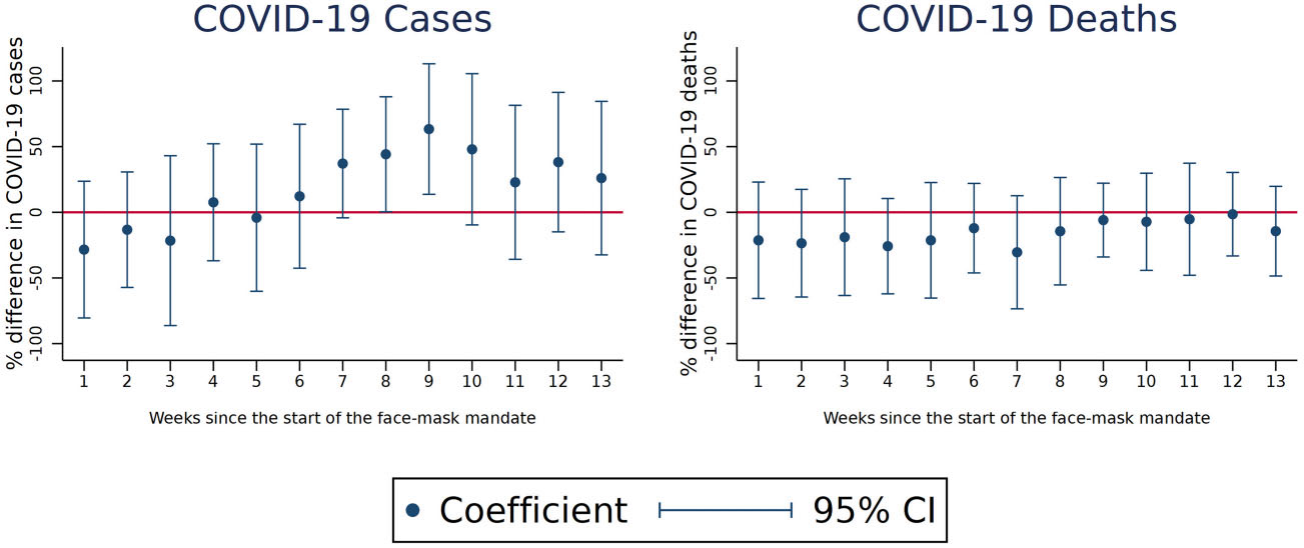
Effect of the face-mask mandate on COVID-19 cases and deaths over time *Note*: The dots indicate the estimated *β*_2_ in week *w*; weighted using population as analytical weights. The percentage difference is approximated by the log. Week 1 is the first week after the treatment, until the 13th week. Outcome defined as Supplementary equations (S4) and (S5), respectively. Each vertical bar represents the respective 95% confidence interval.

We were unable to confidently establish the effect of the face-mask mandate on COVID-19 cases and deaths given the wide 95% CIs in figure 3. However, while none of these effect estimates reached statistical significance, the pattern of the point estimates shown in figure 3 suggests that the face-mask mandate may have decreased COVID-19 deaths in the initial 2 months after implementation, with the effect disappearing for longer time horizons. Supplementary text S5, Tables S21 and S22 and figures S15 and S16 provide more details.

### Impact of adding contact tracing and social distancing on all-cause mortality

We assessed the effect of adding contact tracing and/or social distancing policies to the face-mask mandate. This analysis does not change our main finding of a null effect of mask mandates. There is some indication that combining face-mask mandates with social distancing rules might have had a moderate impact on all-cause mortality. Please refer to the Supplementary appendix (Supplementary text S4, Supplementary tables S16–S20) for further discussion.

## Discussion

While some of our 95% CIs are wide and can thus not exclude the possibility of substantial effects of the face-mask mandate, in combination, all our analyses suggest that the face-mask mandate did not have large effects on all-cause mortality. For instance, the lower bound for a beneficial effect of the face-mask mandate on all-cause mortality that is compatible with our 95% CI in our primary analysis approach is a 3.4% reduction in the weekly number of deaths in a canton. There are several possible reasons for why we did not find substantial mortality-reducing effects of the mandate. First, although the consistency of our estimates across analytical approaches increases our confidence in the findings, we cannot exclude the possibility of important confounding. Confounding would occur if covariates (e.g., COVID-19 mitigating behaviour) change differently over time in the treated and control cantons or have a time-varying effect on the outcome. Second, the population may have begun to adopt face-mask wearing prior to any official face-mask mandate. Third, the population may not have adhered to the mandate. Last but not least, the population may have adopted other measures (e.g., refraining from frequenting public indoor spaces) to reduce their risk of SARS-CoV-2 infection, such that there was no substantial additional benefit gained from face-mask wearing.

Although there likely is substantial variation by face-mask type (e.g. cloth masks versus surgical masks or N95 masks), there is strong evidence that face masks are effective in reducing symptomatic SARS-CoV-2 and other infections.^3–6^^,^^17–20^ However, the evidence on the impact of face-mask mandates is far weaker. In the USA, Chernozhukov *et al*.^5^ exploited variation in policy implementation during the early stages of the COVID-19 pandemic between states to estimate that face-mask mandates for employees in public businesses led to large reductions in COVID-19 deaths. Similarly, Lyu and Wehby^6^ adopted an event-study approach that found that state government face-mask mandates led to a reduction in new COVID-19 cases at the county level between 31 March and 22 May 2020. Two studies from Germany^9^^,^^10^ took advantage of regional variation in the timing of face-mask mandates and estimated a substantial effect on reducing new COVID-19 cases. Finally, a Canadian study,^8^ taking advantage of variation in the start of face-mask mandates between public health regions in Ontario, estimated that such mandates led to a reduction of COVID-19 cases by 25% during the first few weeks of implementation.

In the absence of sufficiently powered randomized controlled trials in Europe, our study is not only an important contribution to this emerging literature because of its focus on a new setting (Switzerland) but, most importantly, because it examines all-cause mortality as its primary outcome. Measuring COVID-19 cases and deaths has several difficulties, which are avoided by focusing on all-cause mortality. For instance, COVID-19 case identification is dependent on the extent to which a country conducts testing and the population’s willingness to undergo or seek out tests.^21^ Similarly, reliably assigning the cause of death to COVID-19 can be difficult if diagnostic codes change over time or individuals die from a combination of proximal causes.^22^^,^^23^ In addition, focusing on COVID-19 deaths ignores indirect effects of face-mask mandates, such as deaths that are averted because the mandate improved the ability of the health system to care for patients without COVID-19 by preventing the health system from being overwhelmed with COVID-19 patients.

Our study has several additional strengths. First and foremost, our analysis is based on a set of reliable administrative data. We constructed a panel containing data on the weekly number of deaths in each Swiss canton between 2012 and 2020. We also added data on total population between 2011 and 2019. Second, we used several different quasi-experimental techniques to assess the robustness of our findings. Third, we set the post-policy period from the date on which the face-mask mandate was introduced in the first canton (on 7 July). This allowed us to control for the possibility of policy anticipation, which in turn may affect people’s behaviour (e.g. voluntary adoption of face-mask wearing prior to the policy coming into effect). Fourth, with long data series on deaths, we can control for canton and time fixed effects (week of the year, for example) and thus allow for a flexible pre-trend in the outcome variables. This characteristic of our dataset also allowed us to examine heterogeneity in the effect of the face-mask mandate over time. Lastly, the quasi-experimental setting in Switzerland allowed us to not only study the effect of face-mask mandates but also the combination of face-mask mandates with contact tracing and social-distancing rules.

However, our study also has several caveats. First, our study is ecologic in nature. While we do not expect significant temporal changes in cantons’ populations that might distort our estimations, we want to caution against drawing conclusions from our policy analysis of face-mask mandates about the effectiveness of face-mask wearing per se. Second, one might be concerned that cantons that expect to benefit more from a face-mask mandate select into introducing such a mandate. However, if that were the case, it would lead to an overestimation of the benefit of the mandate. Our study, however, found a null effect. Third, face-mask mandates might have effects only several days after their implementation. We address this issue by restricting our study timeframe to allow for a sufficient follow-up time and by estimating dynamic treatment effects. Fourth, we can only test for the additional effect of imposing compulsory mask wearing in public places beyond mandating face-mask wearing on public transportation. Fifth, the effects of face-mask mandates may be different in settings other than Switzerland for numerous reasons, including variation in the characteristics of the epidemic, the population, and the policy design and implementation. Sixth, the effects of face-mask mandates may be different at this current stage of the epidemic, such as because of differences in the circulating SARS-CoV-2 variants, the type of face masks that are worn, or because the population is more accustomed to face-mask wearing.

In conclusion, using a variety of statistical approaches, we took advantage of the quasi-experimental policy environment in Switzerland to study the effect of mandating face-mask wearing in all indoor public spaces (in addition to mandating face masks on public transportation) in mid-to-late 2020 on all-cause mortality. We did not detect statistically significant impacts of this policy, neither on all-cause mortality, nor on COVID-19 cases and deaths. The statistical power of our analysis was sufficient to conclude that large positive or negative effects of the face-mask mandates on all-cause mortality are unlikely. The 95% CI for our primary analysis approach ranged from a relative change in all-cause mortality of −3.4% to 2.7%. We also did not find any evidence for substantial effect heterogeneity by sex, age or time since implementation of the policy. There was some suggestion that the combination of face-mask mandates with social distancing rules might have been moderately effective.

## Supplementary data

Supplementary data are available at *EURPUB* online.

## Funding

P.G. was supported by a New Innovator Award (1DP2AI171011-01) from The National Institute of Allergy and Infectious Diseases. P.G. is a Chan Zuckerberg Biohub investigator.

## Role of funder

The funders had no role in the study design; in the collection, analysis, and interpretation of data; in the writing of the report; and in the decision to submit the article for publication.

*Conflicts of interest*: None declared.

## Data sharing

Data are all publicly available. Information on how to access the data from the Swiss National Statistical Office is provided in the manuscript. Statistical code to reproduce the results of this paper is available at https://github.com/FelixMichalik/Face-Masks-Mandates-Switzerland.

Key points

- Heterogeneity in face-mask mandate policies across cantons in Switzerland in mid-to-late 2020 provided an opportunity for the estimation of their effect on mortality.
- Mandating face-mask use in public indoor spaces in Switzerland does not appear to have resulted in large reductions in all-cause mortality in the short term.
- We did not find any evidence for substantial effect heterogeneity by sex, age or time since the implementation of the policy.
- We found suggestive evidence that combining face-mask mandates with social distancing rules reduced all-cause mortality.

## Supplementary Material

ckac123_Supplementary_Data
