# Supplementary material for: The impact of face-mask mandates on all-cause mortality in Switzerland: a quasi-experimental study
Source: Eur J Public Health. 2022 Sep 10;32(5):818–24. doi: 10.1093/eurpub/ckac123 (PMC9527954; doi:10.1093/eurpub/ckac123)
Supplement: ckac123_Supplementary_Data [file ckac123_supplementary_data.zip › ejph-2022-02-om-0080-File002.docx]

**Supplementary appendix**

[Text S1. Policy Timeline 3](#_Toc108619808)

[Figure S1. Summary of Policy Timeline from July to August 2020 8](#_Toc108619809)

[Figure S2. Summary of Policy Timeline from September to October 2020 8](#_Toc108619810)

[Text S2. Definition of main outcome: all-cause mortality 9](#_Toc108619811)

[Table S1. Descriptive Statistics by Sex and Age Category at the National Level 10](#_Toc108619812)

[Table S2. Descriptive Statistics by Sex and Age Category for each Canton 11](#_Toc108619813)

[Table S3. Difference-in-Difference regression on all-cause mortality. Age-class: 0-29 12](#_Toc108619814)

[Table S4. Difference-in-Difference regression on all-cause mortality. Age-class: 30-59 13](#_Toc108619815)

[Table S5. Difference-in-Difference regression on all-cause mortality. Age-class: 60-90 13](#_Toc108619816)

[Table S6. Difference-in-Difference regression on all-cause mortality. Age-class: 90+ 14](#_Toc108619817)

[Table S7. Pooled Difference-in-Difference regression on male all-cause mortality 15](#_Toc108619818)

[Table S8. Pooled Difference-in-Difference regression on female all-cause mortality 16](#_Toc108619819)

[Table S9: Pooled Difference-in-Difference regression on total all-cause mortality 17](#_Toc108619820)

[Figure S3: Event Study. All-Cause Mortality of Males by Age-Class 18](#_Toc108619821)

[Figure S4: Event Study. All-Cause Mortality of Females by Age-Class 19](#_Toc108619822)

[Figure S5: Event Study. All-Cause Mortality of Total Population by Age-Class 20](#_Toc108619823)

[Figure S6: Difference-in-Difference Regression on Male All-Cause Mortality with Dynamic Beta, by Age Class 21](#_Toc108619824)

[Figure S7: Difference-in-Difference Regression on Female All-Cause Mortality with Dynamic Beta, by Age Class 22](#_Toc108619825)

[Figure S8: Difference-in-Difference Regression on Total All-Cause Mortality with Dynamic Beta, by Age Class 23](#_Toc108619826)

[Table S10: Pooled regression on sex 24](#_Toc108619827)

[Text S3: Staggered Difference-in-Differences Model 25](#_Toc108619828)

[Table S11: Staggered Difference-in-Difference 25](#_Toc108619829)

[Table S12: Staggered Difference-in-Difference: age 0-29 25](#_Toc108619830)

[Table S13: Staggered Difference-in-Difference: age 30-59 26](#_Toc108619831)

[Table S14: Staggered Difference-in-Difference: age 60-90 26](#_Toc108619832)

[Table S15: Staggered Difference-in-Difference: age 90+ 27](#_Toc108619833)

[Text S4. Impact of Adding Contact Tracing and Social Distancing to Face-Mask Mandates 28](#_Toc108619834)

[Table S16. Policy mix 30](#_Toc108619835)

[Table S17: Policy Mix regression: age 0-29 31](#_Toc108619836)

[Table S18: Policy Mix regression: age 30-59 32](#_Toc108619837)

[Table S19: Policy Mix regression: age 60-90 33](#_Toc108619838)

[Table S20: Policy Mix regression: age 90+ 34](#_Toc108619839)

[Figure S9: Pre-trend male log all-cause mortality 35](#_Toc108619840)

[Figure S10: Pre-trend male log all-cause mortality, LOWESS 36](#_Toc108619841)

[Figure S11: Pre-trend female log all-cause mortality 37](#_Toc108619842)

[Figure S12: Pre-trend male log all-cause mortality, LOWESS 38](#_Toc108619843)

[Figure S13: Pre-trend both sexes log all-cause mortality 39](#_Toc108619844)

[Figure S14: Pre-trend both sexes log all-cause mortality, LOWESS 40](#_Toc108619845)

[Text S5: Analysis of covid outcomes 41](#_Toc108619846)

[Table S21: Descriptive Statistics of Covid-19 variables 41](#_Toc108619847)

[Figure S15: Pre-trends of Covid cases share 42](#_Toc108619848)

[Figure S16: Pre-trends of Covid deaths share 42](#_Toc108619849)

[Table S22: Difference-in-Difference regression on covid outcomes 43](#_Toc108619850)

[Table S23: Main Difference in differences table – unweighted 44](#_Toc108619851)

[Figure S17: Event study – unweighted 45](#_Toc108619852)

[Figure S18: Difference-in-Difference regression with dynamic beta – unweighted 46](#_Toc108619853)

[Figure S19: Difference-in-Difference regression on total all-cause mortality with dynamic beta, by age class – unweighted 47](#_Toc108619854)

[Figure S20: Difference-in-Difference regression on covid cases and deaths with dynamic beta – unweighted 48](#_Toc108619855)

[Text S6: Treatment effect heterogeneity 49](#_Toc108619856)

[Appendix References 49](#_Toc108619857)

# Text S1. Policy Timeline

Over the first wave of the pandemic, Covid-19 containment policies in Switzerland were enacted on two levels. Federal policies, imposed by the Federal Council, were valid in the whole country. While Cantonal policies, decided by Cantonal Authorities, applied to the specific canton only (Federal Office of Public Health, 2021).

**Federal Policies:** On March 16, 2020, the Federal Council declared Switzerland to be in an ‘Extraordinary Situation’, imposing a partial lock-down.^^[[1]](#footnote-1)^^ After a month the Federal Government announced an easing of the restrictions in small steps between April and July. Hence, between April 27 and the end of June, most of the businesses, bars and restaurants reopened. People were always required to leave their contact information in written form (e.g. name and telephone number). Primary and lower secondary schools as well as higher levels of education in groups of 5 - were resumed. Starting by June 6 clubs, cinemas and all other leisure activities were restored. Also, events with a number of participants between 300 and 1000 were allowed again. In the whole country, as of July 6 wearing a face masks became compulsory in public transportation and quarantine rules were introduced for travelers coming from regions with high risk of infection.^^[[2]](#footnote-2)^^ Finally, on [October 18, 2020 the Federation introduced a mandate of face-mask wearing in all public indoor spaces (e.g. supermarkets, train stations, libraries, shops).](https://www.bag.admin.ch/bag/en/home/krankheiten/ausbrueche-epidemien-pandemien/aktuelle-ausbrueche-epidemien/novel-cov/massnahmen-des-bundes.html)

[By the beginning of July some of the cantons started to impose their own policies on NPIs, including distancing, tracing and stricter requirements on face-mask wearing. Some of the cantons did not take additional measures beyond those imposed by the Federation in that entire period (Appenzell Ausserrhoden, Appenzell Innerrhoden, Glarus, Nidwalden, Obwalden, Switz, Uri).](https://www.bag.admin.ch/bag/en/home/krankheiten/ausbrueche-epidemien-pandemien/aktuelle-ausbrueche-epidemien/novel-cov/massnahmen-des-bundes.html)

[**Aargau:**](https://www.bag.admin.ch/bag/en/home/krankheiten/ausbrueche-epidemien-pandemien/aktuelle-ausbrueche-epidemien/novel-cov/massnahmen-des-bundes.html) [From July 3 onward, the canton has required clubs, bars and restaurants to gather contact details of their guests. By July 9, the canton has limited the maximum number of guests in restaurants and events to 100.](https://www.bag.admin.ch/bag/en/home/krankheiten/ausbrueche-epidemien-pandemien/aktuelle-ausbrueche-epidemien/novel-cov/massnahmen-des-bundes.html)

[**Basel-Landschaft**](https://www.bag.admin.ch/bag/en/home/krankheiten/ausbrueche-epidemien-pandemien/aktuelle-ausbrueche-epidemien/novel-cov/massnahmen-des-bundes.html) [and](https://www.bag.admin.ch/bag/en/home/krankheiten/ausbrueche-epidemien-pandemien/aktuelle-ausbrueche-epidemien/novel-cov/massnahmen-des-bundes.html) [**Basel-Stadt:**](https://www.bag.admin.ch/bag/en/home/krankheiten/ausbrueche-epidemien-pandemien/aktuelle-ausbrueche-epidemien/novel-cov/massnahmen-des-bundes.html) [From July 6 onward the cantons have required clubs, bars and restaurants to gather contact details of their guests. By July 9, the cantons have limited the maximum number of guests in restaurants and events to 100. In addition, on August 24](https://www.bag.admin.ch/bag/en/home/krankheiten/ausbrueche-epidemien-pandemien/aktuelle-ausbrueche-epidemien/novel-cov/massnahmen-des-bundes.html) [**Basel-Stadt**](https://www.bag.admin.ch/bag/en/home/krankheiten/ausbrueche-epidemien-pandemien/aktuelle-ausbrueche-epidemien/novel-cov/massnahmen-des-bundes.html) [made face-mask mandatory in shops and for restaurant employees.](https://www.bag.admin.ch/bag/en/home/krankheiten/ausbrueche-epidemien-pandemien/aktuelle-ausbrueche-epidemien/novel-cov/massnahmen-des-bundes.html)

[**Bern:**](https://www.bag.admin.ch/bag/en/home/krankheiten/ausbrueche-epidemien-pandemien/aktuelle-ausbrueche-epidemien/novel-cov/massnahmen-des-bundes.html) [From July 17 onward the canton has required clubs, bars and restaurants to gather contact details of their guests. By October 12, the canton has limited the maximum number of guests in restaurants and events to 100. In addition, customers must consume food and drinks while sitting at their tables. On October 12 Bern made face-mask mandatory in all public indoor spaces.](https://www.bag.admin.ch/bag/en/home/krankheiten/ausbrueche-epidemien-pandemien/aktuelle-ausbrueche-epidemien/novel-cov/massnahmen-des-bundes.html)

[**Fribourg:**](https://www.bag.admin.ch/bag/en/home/krankheiten/ausbrueche-epidemien-pandemien/aktuelle-ausbrueche-epidemien/novel-cov/massnahmen-des-bundes.html) [From July 17 onward the canton has required clubs, bars and restaurants to gather contact details of their guests. In addition, on August 24, Fribourg made face-mask mandatory in shops and restaurants, for both guests and employees.](https://www.bag.admin.ch/bag/en/home/krankheiten/ausbrueche-epidemien-pandemien/aktuelle-ausbrueche-epidemien/novel-cov/massnahmen-des-bundes.html)

[**Geneva:**](https://www.bag.admin.ch/bag/en/home/krankheiten/ausbrueche-epidemien-pandemien/aktuelle-ausbrueche-epidemien/novel-cov/massnahmen-des-bundes.html) [From July 24 onward the canton has required clubs, bars and restaurants to gather contact details of their guests. In addition, on July 24 Geneva made face-mask mandatory in restaurants, for employees only. The canton extended the requirement to shops on July 28, and to bars and restaurants guests on July 31. Starting from July 31 clubs have closed. From August 18 onward the canton has required bars and restaurants to gather contact details of their guests. Further, private events were allowed up to 100 attendees, public events of up to 1000 participants had to be divided into sections with up to 100 people each. Finally, on October 14, Geneva made face-mask mandatory in all public places.](https://www.bag.admin.ch/bag/en/home/krankheiten/ausbrueche-epidemien-pandemien/aktuelle-ausbrueche-epidemien/novel-cov/massnahmen-des-bundes.html)

**Grischun:** On October 17, the canton made face-mask mandatory in all public indoor

spaces.

**Jura:** On July 7, the canton made face-mask mandatory in shops. From August 25 onward the canton has required clubs, bars, and restaurants to gather contact details of their guests. The canton extended the face-mask requirement to restaurants staff and customers and spectators of indoor events on October 9. By the same date, the canton has limited the maximum number of people in private events to 100. Since October 14 these measures have also applied to private events and tracing of guests must be done

electronically.

**Luzern:** From July 4 onward the canton has required clubs, bars and restaurants to gather contact details of their guests. By July 17, the canton has limited the maximum number of guests in restaurants and events to 100. Public events of up to 1000 participants had to be divided into sections with up to 100 people each and information of attendees had to be gathered. On October 17 Luzern made face-mask mandatory in all public indoor spaces.

**Neuchâtel:** On August 21, the canton made face-mask mandatory in shops (except if less than 10 people were in the shop). By the same date, the canton has limited the maximum number of guests in restaurants and events to 100.

**Schaffhausen:** From July 10 onward the canton has required clubs, bars and restaurants to gather contact details of their guests. On October 16, Schaffhausen made face-mask mandatory in shops and supermarkets.

**Solothurn:** From July 3 onward the canton has required clubs, bars and restaurants to gather contact details of their guests. By July 9 the canton has limited the maximum number of guests in restaurants and events to 100. On September 3 Solothurn made face-mask mandatory in all shops.

**St. Gallen:** From September 25 onward the canton has required clubs, bars and restaurants to gather contact details of their guests.

**Thurgau:** From August 14 onward the canton has required clubs, bars and restaurants to gather contact details of their guests.

**Ticino:** By July 9 the canton has limited the maximum number of people in public spaces to 100. On October 9 all clubs have closed. On October 10, Ticino made facemask mandatory in shops. From the same date onward the canton has required clubs, bars and restaurants to gather contact details of their guests. At a time, restaurants’ guests have been allowed to drink and eat while seated.

**Valais:** By July 16 the canton has limited the maximum number of guests in restaurants and events to 100 (after 8 PM). From the same date onward the canton has required clubs, bars and restaurants to gather contact details of their guests. On August 31, Valais made face-mask mandatory in all shops.

**Vaud:** On July 8 the canton made face-mask mandatory in all shops providing that there were more than 10 people. From the same date onward the canton has required clubs, bars and restaurants to gather contact details of their guests. On September 17 the canton extended the face-mask requirement to restaurants’ staff and customers. At once Vaud introduced a number of additional measures: all clubs had to close, the maximum number of guests in restaurants and events was limited to 100, while for events with more than 50 people masks were mandatory (organizers must also keep a list of participants). Finally, from the same date onward the canton has required clubs, bars and restaurants to gather contact details of their guests.

**Zug:** From July 13 onward the canton has required clubs, bars and restaurants to gather contact details of their guests. By July 13 the canton has limited the maximum number of guests in restaurants and events to 30. On August 22 Zug introduced a number of additional measures: events with more than 100 attendees were only allowed if distancing could be maintained or masks worn. At the same time measures for bars and clubs were relaxed (100 people were allowed indoors). On October 10, Zug made a face-mask mandatory in shops, supermarkets and restaurants.

**Zürich:** From July 3 onward the canton has required clubs and bars to gather contact details of their guests. The requirement was extended to restaurants from August 27. By the same date, the canton made face-mask mandatory in all shops and supermarkets and also limited the maximum number of guests in restaurants and events to 100 (rising to 300 provided that an outdoor area was allowed). For private events (e.g., weddings), concerts or church events with more than 100 participants the mask mandate applied. As of September 24 restaurants, bars, and clubs, in which consumption was not necessarily done while seated, were allowed to host up to 300 guests again, as long as face masks were kept. Moreover, prostitutes were required to collect and verify the contact details of their customers. On October 15, Zürich made face-mask mandatory in all restaurants, bars and clubs.

# Figure S1. Summary of Policy Timeline from July to August 2020

**
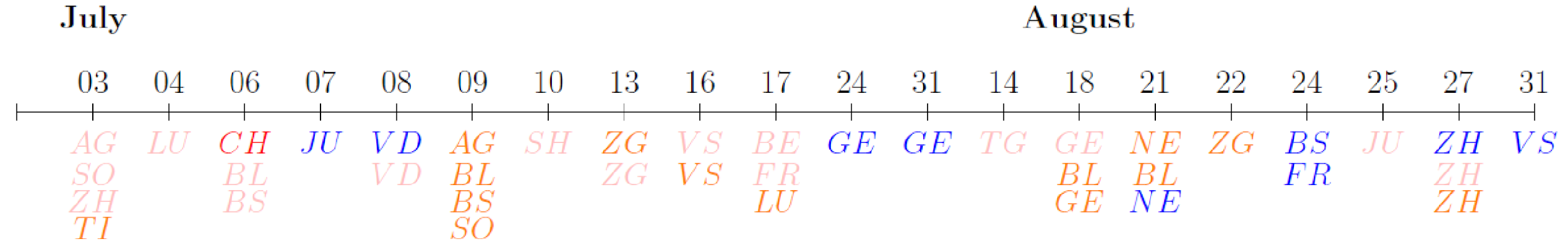
**

# Figure S2. Summary of Policy Timeline from September to October 2020


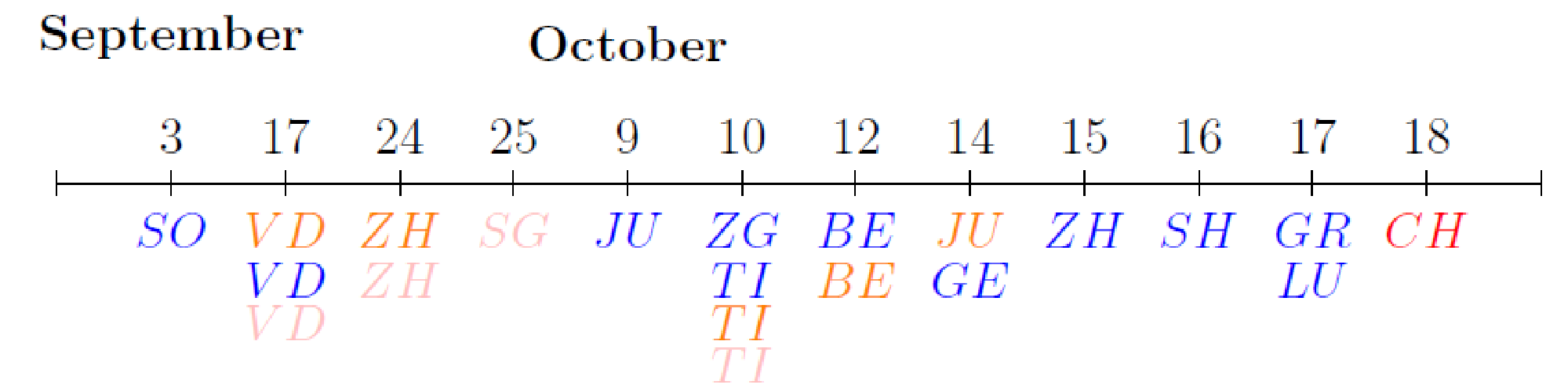


Note: Policies are grouped in 4 categories. In red mask requirements imposed by the Federation, involving all the cantons, are reported. In pink **tracing policies** are reported. In orange **social distancing policies** (e.g., by limiting the number of people to 1000 in public events, 100 or 10 in private ones) are reported. In blue **mask policies** are reported.

# Text S2. Definition of main outcome: all-cause mortality

Our main outcome of interest is all-cause mortality, which we defined as:

$Log(Y_{cwts}) =Log(\frac{\sum_{a=1}^{4} Dea{ths}_{cwts}}{\sum_{a=1}^{4} Popu{lation}_{c\left( t-1 \right)s}}*100,000)$ (Equation S1)

where, *Y_cwts_* represents the all-cause mortality in canton *c*, in week *w* and year *t* for sex *s*. The aggregate number of male (female) deaths was weighted by the canton's male (female) population in the previous year (t -1). This adjustment allows us to take into account i) the different population sizes of cantons, ii) cantonal demographic characteristics, and iii) growth and aging of the population over time.

When examining how effects vary by age and sex, we computed four different outcomes for each age category and sex as follows:

$Log(Y_{cwtsa}) =Log(\frac{Deaths_{cwtsa}}{{Population}_{c(t-1)sa}}*100,000)$ (Equation S2)

Now, *Y_cwtsa_* represents the deaths per population in canton *c*, in week *w* and year *t*, for a specific sex *s* and age cohort *a*.

# Table S1. Descriptive Statistics by Sex and Age Category at the National Level

|  | (1) | (2) | (3) |
| --- | --- | --- | --- |
| VARIABLES | Male | Female | Total |
|  |  |  |  |
| Deaths | 2.64 | 2.68 | 3.32 |
|  | (1.14) | (1.17) | (1.18) |
| Population | 11.47 | 11.47 | 12.13 |
|  | (1.10) | (1.12) | (1.13) |
| All-cause Mortality | 2.69 | 2.72 | 2.71 |
|  | (0.38) | (0.39) | (0.32) |
| Deaths (0-29) | 0.29 | 0.19 | 0.43 |
|  | (0.44) | (0.36) | (0.54) |
| Population (0-29) | 11.13 | 11.19 | 11.73 |
|  | (0.87) | (0.86) | (0.88) |
| All-cause Mortality (0-29) | 0.67 | 0.52 | 0.21 |
|  | (0.79) | (0.81) | (0.76) |
| Deaths (30-59) | 0.90 | 0.64 | 1.19 |
|  | (0.76) | (0.65) | (0.86) |
| Population (30-59) | 11.06 | 11.16 | 11.64 |
|  | (0.92) | (0.90) | (0.97) |
| All-cause Mortality (30-59) | 1.35 | 0.99 | 1.06 |
|  | (0.66) | (0.70) | (0.60) |
| Deaths (60-90) | 2.33 | 2.21 | 2.90 |
|  | (1.11) | (1.11) | (1.16) |
| Population (60-90) | 9.98 | 10.13 | 10.69 |
|  | (1.05) | (1.08) | (1.10) |
| All-cause Mortality (60-90) | 3.86 | 3.60 | 3.72 |
|  | (0.40) | (0.41) | (0.35) |
| Deaths (90+) | 1.24 | 1.79 | 2.09 |
|  | (0.89) | (1.03) | (1.10) |
| Population (90+) | 6.40 | 7.22 | 7.48 |
|  | (0.99) | (1.06) | (1.08) |
| All-cause Mortality (90+) | 6.35 | 6.08 | 6.12 |
|  | (0.61) | (0.50) | (0.46) |
|  |  |  |  |
| Observations | 9,168 | 9,178 | 9,329 |

Note: Summary statistics of the main variables for Switzerland. Each row contains the mean of the variable for male, female, and total population. Standard deviation in parenthesis. Observations contain (i) weekly data of log(deaths) for the first 40 weeks of each year between 2012 and 2020 (ii) yearly data of log population between 2011 and 2019. All-cause Mortality is the log ratio between total deaths in year t and population divided by 100,000 in t – 1.

# Table S2. Descriptive Statistics by Sex and Age Category for each Canton

| Canton | Male | Female | Total | Canton | Male | Female | Total |
| --- | --- | --- | --- | --- | --- | --- | --- |
| AG | 2.615 | 2.618 | 2.622 | OW | 2.571 | 2.516 | 2.5 |
|  | (0.179) | (0.177) | (0.142) |  | (0.573) | (0.578) | (0.527) |
| AI | 2.95 | 2.998 | 2.717 | SG | 2.653 | 2.717 | 2.693 |
|  | (0.489) | (0.501) | (0.566) |  | (0.191) | (0.217) | (0.163) |
| AR | 2.701 | 2.783 | 2.776 | SH | 2.765 | 2.832 | 2.843 |
|  | (0.516) | (0.521) | (0.393) |  | (0.432) | (0.461) | (0.309) |
| BE | 2.84 | 2.886 | 2.867 | SO | 2.764 | 2.835 | 2.813 |
|  | (0.133) | (0.152) | (0.12) |  | (0.243) | (0.25) | (0.183) |
| BL | 2.77 | 2.772 | 2.783 | SZ | 2.573 | 2.54 | 2.585 |
|  | (0.229) | (0.239) | (0.169) |  | (0.358) | (0.356) | (0.259) |
| BS | 2.894 | 3.086 | 3.013 | TG | 2.605 | 2.626 | 2.63 |
|  | (0.286) | (0.251) | (0.197) |  | (0.26) | (0.279) | (0.203) |
| FR | 2.52 | 2.49 | 2.52 | TI | 2.807 | 2.842 | 2.832 |
|  | (0.271) | (0.265) | (0.199) |  | (0.239) | (0.242) | (0.207) |
| GE | 2.521 | 2.588 | 2.563 | UR | 2.788 | 2.778 | 2.773 |
|  | (0.217) | (0.205) | (0.17) |  | (0.582) | (0.573) | (0.48) |
| GL | 2.752 | 2.809 | 2.8 | VD | 2.598 | 2.628 | 2.618 |
|  | (0.584) | (0.571) | (0.459) |  | (0.178) | (0.187) | (0.154) |
| GR | 2.754 | 2.801 | 2.797 | VS | 2.732 | 2.669 | 2.712 |
|  | (0.293) | (0.289) | (0.209) |  | (0.246) | (0.269) | (0.206) |
| JU | 2.781 | 2.782 | 2.824 | ZG | 2.354 | 2.445 | 2.445 |
|  | (0.474) | (0.424) | (0.317) |  | (0.453) | (0.43) | (0.29) |
| LU | 2.633 | 2.651 | 2.653 | ZH | 2.605 | 2.685 | 2.649 |
|  | (0.212) | (0.244) | (0.175) |  | (0.136) | (0.138) | (0.117) |
| NE | 2.788 | 2.828 | 2.828 | Total | 2.686 | 2.719 | 2.708 |
|  | (0.303) | (0.304) | (0.221) |  | (0.38) | (0.39) | (0.324) |
| NW | 2.563 | 2.544 | 2.541 |  |  |  |  |
|  | (0.546) | (0.59) | (0.491) |  |  |  |  |

Note: Summary statistics of *All-cause Mortality* for each canton. Each row contains the mean of the variable at an aggregated age-class level (0 to 90+ years old) for a specific sex in the first 40 weeks of the period January 2012 - October 2020. Standard deviation in parenthesis. *All-cause Mortality* is the log ratio between total deaths in year t and population divided by 100,000 in t − 1.

# Table S3. Difference-in-Difference regression on all-cause mortality. Age-class: 0-29

|  | (1) | (2) | (3) | (4) | (5) | (6) | (7) | (8) | (9) |
| --- | --- | --- | --- | --- | --- | --- | --- | --- | --- |
| VARIABLES | Male  (0-29) | Male  (0-29) | Male  (0-29) | Female  (0-29) | Female  (0-29) | Female  (0-29) | Total  (0-29) | Total  (0-29) | Total  (0-29) |
|  |  |  |  |  |  |  |  |  |  |
| Treat | -0.261 | -0.423*** | -0.422*** | -0.298 | -0.523*** | -0.517*** | -0.138 | -0.166*** | -0.165*** |
|  | (0.216) | (0.005) | (0.005) | (0.259) | (0.003) | (0.004) | (0.157) | (0.002) | (0.003) |
| Post | 0.070 | 0.058 | 0.053 | -0.025 | -0.112** | -0.117 | 0.040 | -0.003 | 0.004 |
|  | (0.049) | (0.039) | (0.039) | (0.102) | (0.042) | (0.075) | (0.048) | (0.035) | (0.036) |
| DiD | -0.064 | -0.081 | -0.091 | 0.035 | 0.139* | 0.139 | 0.002 | 0.044 | 0.034 |
|  | (0.141) | (0.140) | (0.145) | (0.120) | (0.076) | (0.088) | (0.056) | (0.048) | (0.049) |
| Constant | 0.408*** | 0.267*** | 0.389*** | 0.250 | 0.171*** | 0.180*** | -0.068 | -0.236*** | -0.137** |
|  | (0.142) | (0.001) | (0.083) | (0.178) | (0.001) | (0.060) | (0.114) | (0.001) | (0.060) |
|  |  |  |  |  |  |  |  |  |  |
| Observations | 2,975 | 2,975 | 2,975 | 1,883 | 1,883 | 1,883 | 3,854 | 3,854 | 3,854 |
| R-squared | 0.043 | 0.472 | 0.492 | 0.054 | 0.592 | 0.603 | 0.013 | 0.333 | 0.348 |
| Year FE | NO | NO | YES | NO | NO | YES | NO | NO | YES |
| Canton FE | NO | YES | YES | NO | YES | YES | NO | YES | YES |
| Week FE | NO | NO | YES | NO | NO | YES | NO | NO | YES |
| Mean | 0.674 | 0.674 | 0.674 | 0.516 | 0.516 | 0.516 | 0.206 | 0.206 | 0.206 |

Note: Regression results based on Equation (1); weighted using population as analytical weights. Dependent variable defined as the log ratio between deaths in age-class *age* of a specific canton *c* in a week *w* and year *t* and total population of age *age* divided by 100,000 in the canton in year *t-1*. Column 1-2-3 contain observation for male population. Columns 4-5-6 contain observations for female population. Column 7-8-9 contain observations for aggregate male and female population. S.E. clustered at a canton level (*** p<0.01, ** p<0.05, * p<0.1). Period of estimation: between January 2012 and October 4, 2020. *Treated* cantons are those that between July 7 and October 4 have imposed any mask requirement other than Federal indications (e.g., in supermarket, restaurants, open space): BS, FR, GE, JU, NE, SO, VS, VD, ZH. *Post* is equal to 1 for all cantons after July 7.

# Table S4. Difference-in-Difference regression on all-cause mortality. Age-class: 30-59

|  | (1) | (2) | (3) | (4) | (5) | (6) | (7) | (8) | (9) |
| --- | --- | --- | --- | --- | --- | --- | --- | --- | --- |
| VARIABLES | Male  (30-59) | Male  (30-59) | Male  (30-59) | Female (30-59) | Female (30-59) | Female (30-59) | Total  (30-59) | Total  (30-59) | Total  (30-59) |
|  |  |  |  |  |  |  |  |  |  |
| Treat | -0.067 | 0.017*** | 0.017*** | -0.136 | -0.136*** | -0.135*** | -0.064 | -0.037*** | -0.037*** |
|  | (0.060) | (0.003) | (0.003) | (0.098) | (0.002) | (0.002) | (0.046) | (0.002) | (0.002) |
| Post | -0.114*** | -0.109*** | -0.040 | -0.129*** | -0.121*** | -0.054 | -0.126*** | -0.122*** | -0.041 |
|  | (0.028) | (0.028) | (0.051) | (0.042) | (0.042) | (0.055) | (0.040) | (0.040) | (0.051) |
| DiD | -0.019 | -0.019 | -0.020 | 0.039 | 0.038 | 0.033 | -0.032 | -0.035 | -0.037 |
|  | (0.069) | (0.069) | (0.070) | (0.047) | (0.047) | (0.047) | (0.057) | (0.057) | (0.057) |
| Constant | 1.192*** | 1.027*** | 1.048*** | 0.791*** | 0.628*** | 0.630*** | 0.956*** | 0.882*** | 0.935*** |
|  | (0.042) | (0.001) | (0.042) | (0.053) | (0.002) | (0.050) | (0.035) | (0.001) | (0.043) |
|  |  |  |  |  |  |  |  |  |  |
| Observations | 6,501 | 6,501 | 6,501 | 5,424 | 5,424 | 5,424 | 7,349 | 7,349 | 7,349 |
| R-squared | 0.006 | 0.137 | 0.157 | 0.015 | 0.221 | 0.244 | 0.008 | 0.105 | 0.131 |
| Year FE | NO | NO | YES | NO | NO | YES | NO | NO | YES |
| Canton FE | NO | YES | YES | NO | YES | YES | NO | YES | YES |
| Week FE | NO | NO | YES | NO | NO | YES | NO | NO | YES |
| Mean | 1.355 | 1.355 | 1.355 | 0.992 | 0.992 | 0.992 | 1.064 | 1.064 | 1.064 |

Note: See Table S3.

# Table S5. Difference-in-Difference regression on all-cause mortality. Age-class: 60-90

|  | (1) | (2) | (3) | (4) | (5) | (6) | (7) | (8) | (9) |
| --- | --- | --- | --- | --- | --- | --- | --- | --- | --- |
| VARIABLES | Male  (60-90) | Male  (60-90) | Male  (60-90) | Female (60-90) | Female (60-90) | Female (60-90) | Total  (60-90) | Total  (60-90) | Total  (60-90) |
|  |  |  |  |  |  |  |  |  |  |
| Treat | 0.027 | 0.035*** | 0.034*** | 0.006 | 0.051*** | 0.049*** | 0.018 | 0.033*** | 0.032*** |
|  | (0.024) | (0.001) | (0.001) | (0.029) | (0.001) | (0.001) | (0.023) | (0.001) | (0.001) |
| Post | -0.112*** | -0.111*** | -0.005 | -0.165*** | -0.164*** | -0.044* | -0.133*** | -0.133*** | -0.023 |
|  | (0.021) | (0.021) | (0.031) | (0.021) | (0.021) | (0.024) | (0.016) | (0.016) | (0.023) |
| DiD | -0.004 | -0.004 | -0.003 | 0.063** | 0.064** | 0.064** | 0.025 | 0.025 | 0.025 |
|  | (0.025) | (0.025) | (0.025) | (0.026) | (0.026) | (0.026) | (0.019) | (0.018) | (0.019) |
| Constant | 3.859*** | 3.841*** | 3.953*** | 3.587*** | 3.572*** | 3.726*** | 3.732*** | 3.717*** | 3.843*** |
|  | (0.020) | (0.001) | (0.019) | (0.019) | (0.001) | (0.025) | (0.016) | (0.001) | (0.015) |
|  |  |  |  |  |  |  |  |  |  |
| Observations | 9,004 | 9,004 | 9,004 | 8,928 | 8,928 | 8,928 | 9,253 | 9,253 | 9,253 |
| R-squared | 0.009 | 0.055 | 0.137 | 0.009 | 0.061 | 0.152 | 0.013 | 0.078 | 0.213 |
| Year FE | NO | NO | YES | NO | NO | YES | NO | NO | YES |
| Canton FE | NO | YES | YES | NO | YES | YES | NO | YES | YES |
| Week FE | NO | NO | YES | NO | NO | YES | NO | NO | YES |
| Mean | 3.864 | 3.864 | 3.864 | 3.597 | 3.597 | 3.597 | 3.722 | 3.722 | 3.722 |

Note: See Table S3.

# Table S6. Difference-in-Difference regression on all-cause mortality. Age-class: 90+

|  | (1) | (2) | (3) | (4) | (5) | (6) | (7) | (8) | (9) |
| --- | --- | --- | --- | --- | --- | --- | --- | --- | --- |
| VARIABLES | Male 90+ | Male 90+ | Male 90+ | Female 90+ | Female 90+ | Female 90+ | Total  90+ | Total  90+ | Total  90+ |
|  |  |  |  |  |  |  |  |  |  |
| Treat | -0.07* | 0.01** | 0.01** | -0.05 | 0.01*** | 0.00*** | -0.05 | -0.01*** | -0.01*** |
|  | (0.04) | (0.00) | (0.00) | (0.04) | (0.00) | (0.00) | (0.04) | (0.00) | (0.00) |
| Post | -0.17*** | -0.17*** | 0.03 | -0.20*** | -0.19*** | 0.00 | -0.18*** | -0.18*** | 0.01 |
|  | (0.05) | (0.05) | (0.06) | (0.02) | (0.02) | (0.02) | (0.02) | (0.02) | (0.03) |
| DiD | -0.01 | -0.01 | -0.01 | 0.02 | 0.02 | 0.01 | 0.00 | 0.00 | 0.00 |
|  | (0.06) | (0.06) | (0.06) | (0.03) | (0.03) | (0.03) | (0.03) | (0.03) | (0.03) |
| Constant | 6.26*** | 6.24*** | 6.48*** | 6.06*** | 6.09*** | 6.40*** | 6.13*** | 6.16*** | 6.45*** |
|  | (0.02) | (0.00) | (0.03) | (0.02) | (0.00) | (0.02) | (0.02) | (0.00) | (0.02) |
|  |  |  |  |  |  |  |  |  |  |
| Observations | 7,514 | 7,514 | 7,514 | 8,401 | 8,401 | 8,401 | 8,788 | 8,788 | 8,788 |
| R-squared | 0.01 | 0.08 | 0.18 | 0.01 | 0.07 | 0.25 | 0.02 | 0.06 | 0.28 |
| Year FE | NO | NO | YES | NO | NO | YES | NO | NO | YES |
| Canton FE | NO | YES | YES | NO | YES | YES | NO | YES | YES |
| Week FE | NO | NO | YES | NO | NO | YES | NO | NO | YES |
| Mean | 6.35 | 6.35 | 6.35 | 6.08 | 6.08 | 6.08 | 6.12 | 6.12 | 6.12 |

Note: See Table S3.

# Table S7. Pooled Difference-in-Difference regression on male all-cause mortality

|  | (1) | (2) | (3) |
| --- | --- | --- | --- |
| VARIABLES | Model 1 | Model 2 | Model 3 |
|  |  |  |  |
| Post | 0.044 | 0.043 | 0.155*** |
|  | (0.048) | (0.047) | (0.041) |
| Age (60-90) | 3.801*** | 3.780*** | 3.781*** |
|  | (0.130) | (0.138) | (0.137) |
| Age (30-59) | 1.026*** | 1.016*** | 1.016*** |
|  | (0.139) | (0.143) | (0.142) |
| Age (90+) | 6.200*** | 6.182*** | 6.183*** |
|  | (0.128) | (0.135) | (0.134) |
| Did (60-90) | 0.027 | 0.026 | 0.031 |
|  | (0.060) | (0.061) | (0.060) |
| Did (30-59) | -0.029 | -0.029 | -0.024 |
|  | (0.085) | (0.086) | (0.085) |
| Did (90+) | 0.009 | 0.009 | 0.013 |
|  | (0.077) | (0.078) | (0.077) |
| Constant | -0.069 | -0.105 | 0.035 |
|  | (0.117) | (0.109) | (0.114) |
|  |  |  |  |
| Observations | 29,244 | 29,244 | 29,244 |
| R-squared | 0.971 | 0.972 | 0.973 |
| Year FE | NO | NO | YES |
| Canton FE | NO | YES | YES |
| Week FE | NO | NO | YES |
| Did(1)=Did(2) = 0 | 0.839 | 0.849 | 0.852 |
| Did(1)=Did(3) = 0 | 0.639 | 0.655 | 0.631 |
| Did(2)=Did(3) = 0 | 0.716 | 0.728 | 0.686 |

Note: Pooled regression with 4 age-groups: young (0-29); middle (30-59); old (60-90); very old (90+). Baseline group: age 0-29. Models differ in FE. Dependent variable defined as Equation (S2) with male population. *Post* is equal to 1 for all cantons after July 7. S.E. clustered at a canton level (*** p<0.01, ** p<0.05, * p<0.1).

# Table S8. Pooled Difference-in-Difference regression on female all-cause mortality

|  | (1) | (2) | (3) |
| --- | --- | --- | --- |
| VARIABLES | Model 1 | Model 2 | Model 3 |
|  |  |  |  |
| Post | -0.022 | -0.032 | 0.089 |
|  | (0.102) | (0.093) | (0.093) |
| Age (60-90) | 3.344*** | 3.303*** | 3.306*** |
|  | (0.196) | (0.206) | (0.203) |
| Age (30-59) | 0.547*** | 0.532*** | 0.534*** |
|  | (0.152) | (0.154) | (0.152) |
| Age (90+) | 5.825*** | 5.788*** | 5.791*** |
|  | (0.194) | (0.201) | (0.199) |
| Did (60-90) | 0.031 | 0.018 | 0.025 |
|  | (0.113) | (0.104) | (0.106) |
| Did (30-59) | 0.004 | -0.010 | -0.003 |
|  | (0.140) | (0.129) | (0.132) |
| Did (90+) | -0.010 | -0.025 | -0.019 |
|  | (0.127) | (0.117) | (0.119) |
| Constant | 0.244 | 0.218 | 0.357** |
|  | (0.182) | (0.163) | (0.173) |
|  |  |  |  |
| Observations | 24,636 | 24,636 | 24,636 |
| R-squared | 0.964 | 0.966 | 0.967 |
| Year FE | NO | NO | YES |
| Canton FE | NO | YES | YES |
| Week FE | NO | NO | YES |
| Did(1)=Did(2) = 0 | 0.949 | 0.919 | 0.928 |
| Did(1)=Did(3) = 0 | 0.807 | 0.835 | 0.814 |
| Did(2)=Did(3) = 0 | 0.529 | 0.528 | 0.509 |

Note: Pooled regression with 4 age-groups: young (0-29); middle (30-59); old (60-90); very old (90+). Baseline group: age 0-29. Models differ in FE. Dependent variable defined as Equation (S2) with female population. *Post* is equal to 1 for all cantons after July 7. S.E. clustered at a canton level (*** p<0.01, ** p<0.05, * p<0.1).

# Table S9: Pooled Difference-in-Difference regression on total all-cause mortality

|  | (1) | (2) | (3) |
| --- | --- | --- | --- |
| VARIABLES | Model 1 | Model 2 | Model 3 |
|  |  |  |  |
| Post | 0.074 | 0.073 | 0.178*** |
|  | (0.048) | (0.047) | (0.048) |
| Age (60-90) | 3.452*** | 3.407*** | 3.407*** |
|  | (0.162) | (0.175) | (0.174) |
| Age (30-59) | 0.785*** | 0.768*** | 0.768*** |
|  | (0.151) | (0.155) | (0.154) |
| Age (90+) | 5.861*** | 5.834*** | 5.833*** |
|  | (0.151) | (0.158) | (0.157) |
| Did (60-90) | 0.069 | 0.071 | 0.071 |
|  | (0.142) | (0.142) | (0.143) |
| Did (30-59) | 0.059 | 0.061 | 0.061 |
|  | (0.099) | (0.100) | (0.100) |
| Did (90+) | 0.053 | 0.056 | 0.056 |
|  | (0.181) | (0.182) | (0.181) |
| Constant | 0.407*** | 0.350** | 0.478*** |
|  | (0.145) | (0.134) | (0.131) |
|  |  |  |  |
| Observations | 25,994 | 25,994 | 25,994 |
| R-squared | 0.959 | 0.961 | 0.962 |
| Year FE | NO | NO | YES |
| Canton FE | NO | YES | YES |
| Week FE | NO | NO | YES |
| Did(1)=Did(2) = 0 | 0.780 | 0.770 | 0.767 |
| Did(1)=Did(3) = 0 | 0.838 | 0.831 | 0.827 |
| Did(2)=Did(3) = 0 | 0.789 | 0.789 | 0.787 |

Note: Pooled regression with 4 age-groups: young (0-29); middle (30-59); old (60-90); very old (90+). Baseline group: age 0-29. Models differ in FE. Dependent variable defined as Equation (S2) at aggregate sex level. *Post* is equal to 1 for all cantons after July 7. S.E. clustered at a canton level (*** p<0.01, ** p<0.05, * p<0.1).

# Figure S3: Event Study. All-Cause Mortality of Males by Age-Class


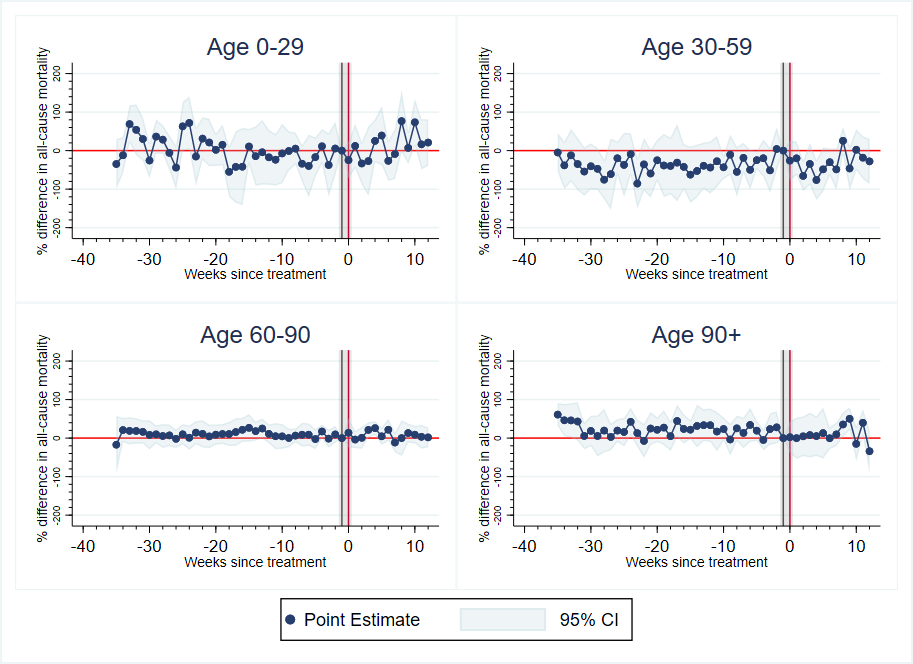


Note: Each panel reports the estimates for a specific age-class; weighted using population as analytical weights. The % difference in all-cause mortality is approximated by the log. Point estimates (blue points) are displayed along with their 95% confidence intervals (light blue area). Baseline period: 1 week prior to the adoption of the face masks policy in each adopting canton, corresponding to the solid vertical line in the plot.

# Figure S4: Event Study. All-Cause Mortality of Females by Age-Class


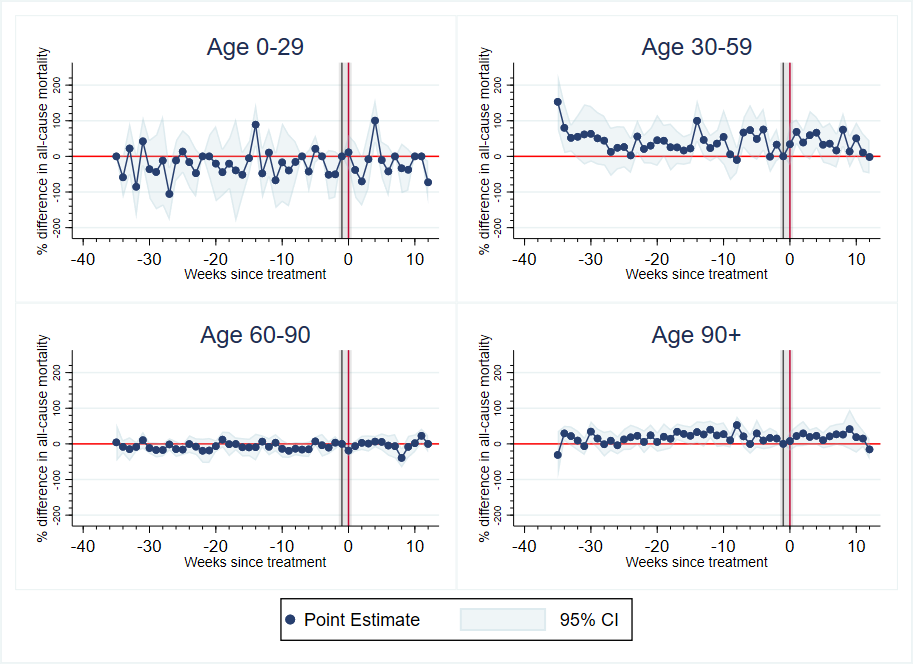


Note: Each panel reports the estimates for a specific age-class; weighted using population as analytical weights. The % difference in all-cause mortality is approximated by the log. Point estimates (blue points) are displayed along with their 95% confidence intervals (light blue area). Baseline period: 1 week prior to the adoption of the face masks policy in each adopting canton, corresponding to the solid vertical line in the plot.

# Figure S5: Event Study. All-Cause Mortality of Total Population by Age-Class


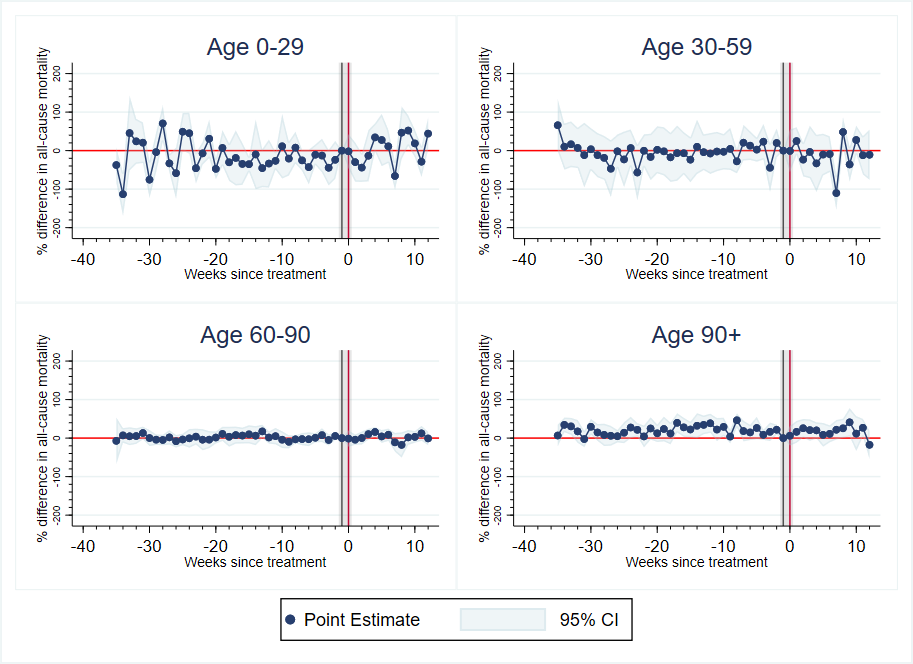


Notes: Each panel reports the estimates for a specific age-class; weighted using population as analytical weights. The % difference in all-cause mortality is approximated by the log. Point estimates (blue points) are displayed along with their 95% confidence intervals (light blue area). Baseline period: 1 week prior to the adoption of the face masks policy in each adopting canton, corresponding to the solid vertical line in the plot.

# Figure S6: Difference-in-Difference Regression on Male All-Cause Mortality with Dynamic Beta, by Age Class


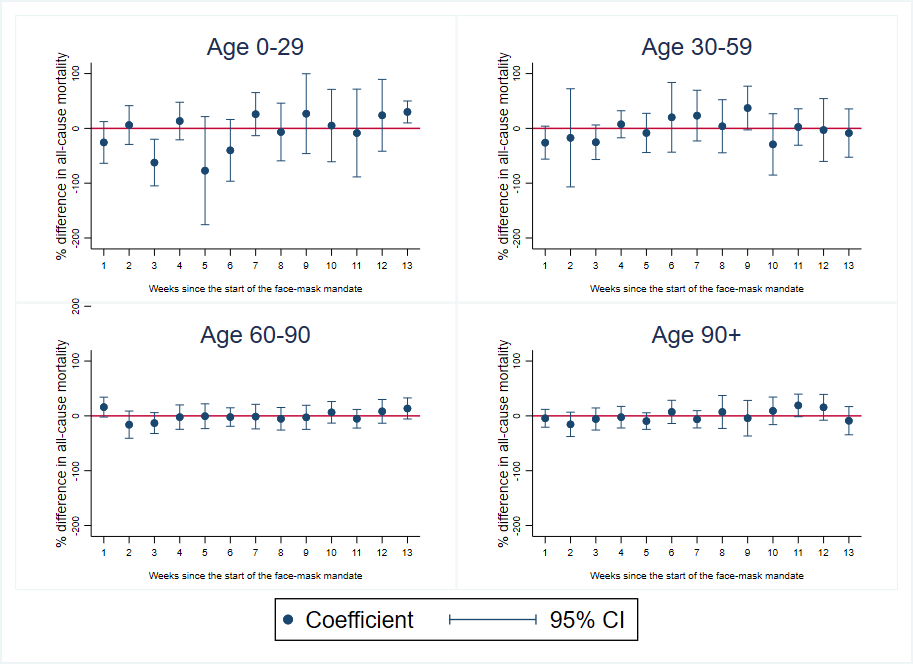


Note: Each panel reports the estimates for a specific age-class; weighted using population as analytical weights. The % difference in all-cause mortality is approximated by the log. Blue dots are the estimated β_2w_ of Equation (S2) in week *w*. Week 1 is the first week after the treatment, until the 13th week after the treatment. Outcome defined as Equation (7). Each bar represents the respective 95% confidence interval.

# Figure S7: Difference-in-Difference Regression on Female All-Cause Mortality with Dynamic Beta, by Age Class


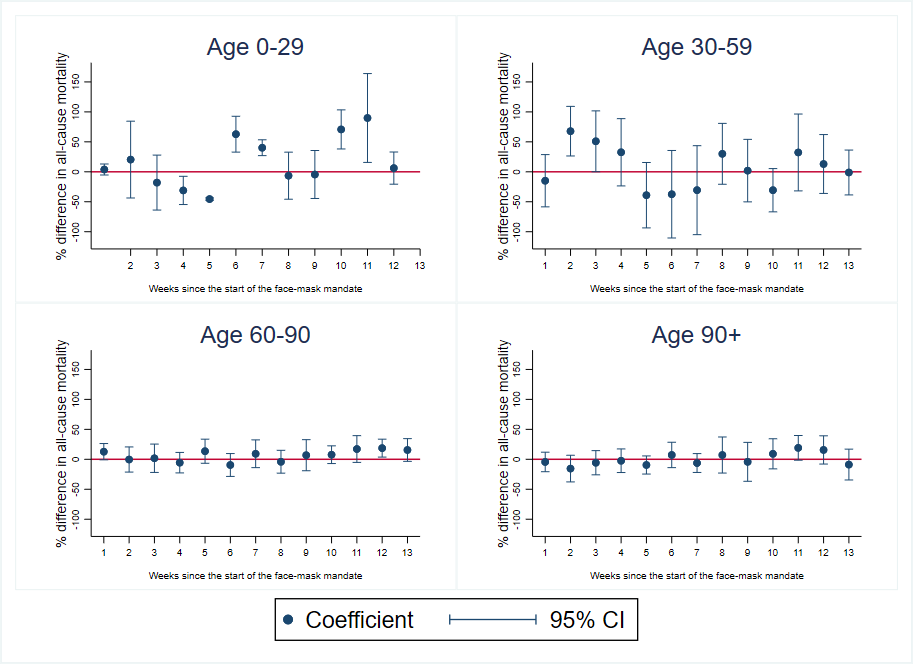


Note: Each panel reports the estimates for a specific age-class; weighted using population as analytical weights. The % difference in all-cause mortality is approximated by the log. Blue dots are the estimated β_2w_ of Equation (7) in week *w*. Week 1 is the first week after the treatment, until the 13th week after the treatment. Outcome defined as Equation (S2). Each bar represents the respective 95% confidence interval.

# Figure S8: Difference-in-Difference Regression on Total All-Cause Mortality with Dynamic Beta, by Age Class


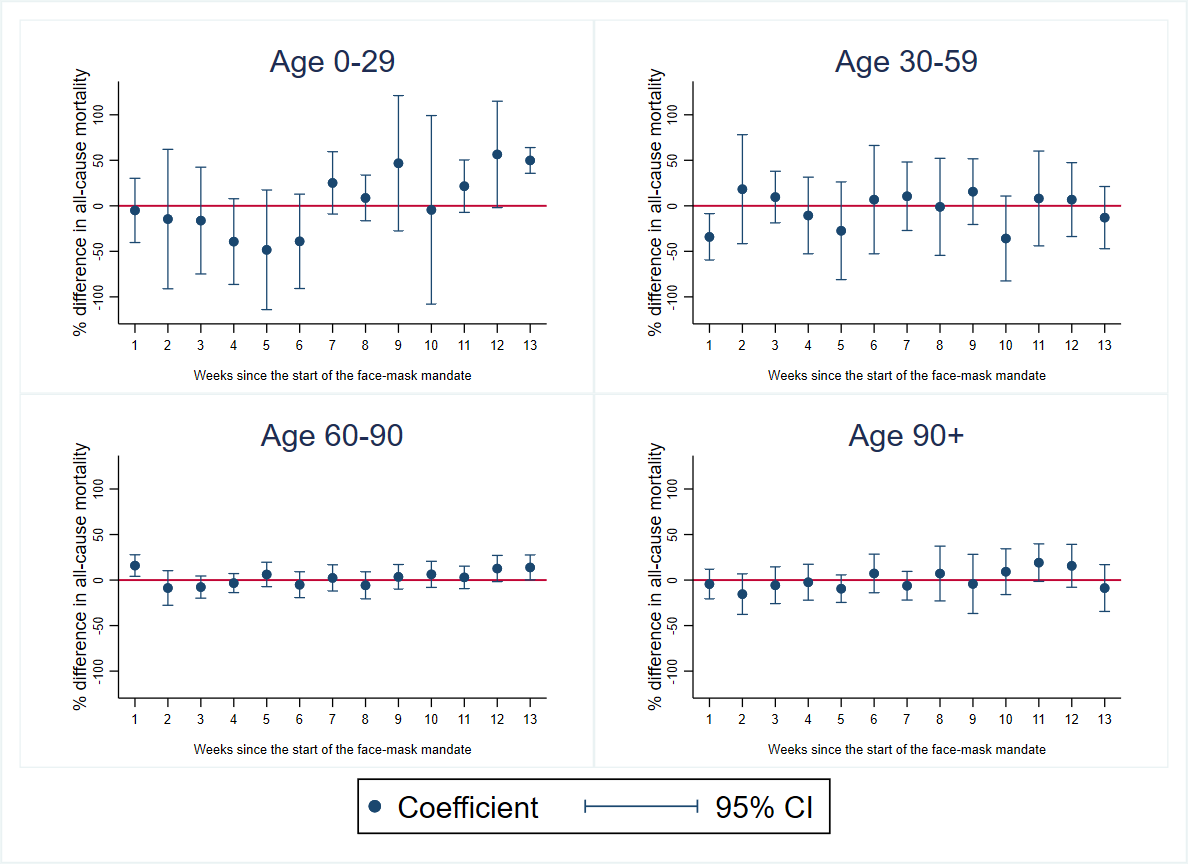


Note: Each panel reports the estimates for a specific age-class; weighted using population as analytical weights. The % difference in all-cause mortality is approximated by the log. Blue dots are the estimated β_2w_ of Equation (7) in week *w*. Week 1 is the first week after the treatment, until the 13th week after the treatment. Outcome defined as Equation (S2). Each bar represents the respective 95% confidence interval.

# Table S10: Pooled regression on sex

|  | (1) | (2) | (3) |
| --- | --- | --- | --- |
| VARIABLES | Model 1 | Model 2 | Model 3 |
|  |  |  |  |
| Post | -0.478*** | -0.479*** | -0.0884 |
|  | (0.0610) | (0.0612) | (0.0836) |
| Treat_M_ | 0.764*** | 0.764*** | 0.764*** |
|  | (0.115) | (0.116) | (0.116) |
| Did | 0.114 | 0.116 | 0.115 |
|  | (0.0938) | (0.0938) | (0.0939) |
| Did_M_ | -0.229 | -0.229 | -0.229 |
|  | (0.214) | (0.214) | (0.214) |
| Constant | 10.18*** | 10.41*** | 10.91*** |
|  | (0.0890) | (0.0579) | (0.0894) |
|  |  |  |  |
| Observations | 18,720 | 18,720 | 18,720 |
| R-squared | 0.086 | 0.247 | 0.291 |
| Year FE | NO | NO | YES |
| Canton FE | NO | YES | YES |
| Week FE | NO | NO | YES |
| Did(M)-Did(F)^a^ = 0 | -0.115 | -0.113 | -0.114 |
| p-value ^b^ | 0.470 | 0.475 | 0.472 |

Note: Pooled regression with 2 sex groups: male and female; weighted using population as analytical weights. Baseline group: female (age 0-90+). Outcome defined as Equation (S1). *Post* is equal to 1 for all cantons after July 7. *Did^M^* is the interaction between *Sex* x *Treat* x *Post*. (a) Parameter estimation of the difference between the 2 coefficients. (b) Significance level at which H_0_: Did_M_ − Did = 0 is rejected. S.E. clustered at a canton level (*** p<0.01, ** p<0.05, * p<0.1).

# Text S3: Staggered Difference-in-Differences Model

We estimate the following equation:

$Log\left( Y_{ct} \right)=\alpha+\beta Mask_{ct}+\gamma_{t}+\delta_{c}+\epsilon_{ct}$ (Equation S3)

# Table S11: Staggered Difference-in-Difference

|  | (1) | (2) | (3) |
| --- | --- | --- | --- |
| VARIABLES | Male | Female | Total |
|  |  |  |  |
| Mask | 0.00370 | 0.0177 | 0.0120 |
|  | (0.0177) | (0.0300) | (0.0197) |
| Constant | 2.752*** | 2.870*** | 2.821*** |
|  | (0.0143) | (0.0167) | (0.0122) |
|  |  |  |  |
| Observations | 9,168 | 9,178 | 9,329 |
| R-squared | 0.121 | 0.195 | 0.248 |
| Number of canton_id | 26 | 26 | 26 |
| Year FE | YES | YES | YES |
| Canton FE | YES | YES | YES |
| Week FE | YES | YES | YES |
| Mean | 2.686 | 2.719 | 2.708 |

Note: Dependent variable defined as Equation (S1); weighted using population as analytical weights. *Mask* is a dummy equal to 1 after the date in which canton *c* is treated, otherwise it is 0. Period of estimation: between January 2012 and October 4^th^, 2020. S.E. clustered at a canton level (*** p<0.01, ** p<0.05, * p<0.1).

# Table S12: Staggered Difference-in-Difference: age 0-29

|  | (1) | (2) | (3) |
| --- | --- | --- | --- |
| VARIABLES | Male (0-29) | Female (0-29) | Total (0-29) |
|  |  |  |  |
| Mask | 0.0487 | 0.109 | 0.140** |
|  | (0.132) | (0.111) | (0.0601) |
| Constant | 0.387*** | 0.0905 | -0.0423 |
|  | (0.0817) | (0.0594) | (0.0626) |
|  |  |  |  |
| Observations | 2,975 | 1,883 | 3,854 |
| R-squared | 0.038 | 0.030 | 0.023 |
| Number of canton_id | 26 | 26 | 26 |
| Year FE | YES | YES | YES |
| Canton FE | YES | YES | YES |
| Week FE | YES | YES | YES |
| Mean | 0.674 | 0.516 | 0.206 |

Note: Dependent variable defined as Equation (S2). For more details see Table S11.

# Table S13: Staggered Difference-in-Difference: age 30-59

|  | (1) | (2) | (3) |
| --- | --- | --- | --- |
| VARIABLES | Male (30-59) | Female (30-59) | Total (30-59) |
|  |  |  |  |
| Mask | -0.0711 | 0.0180 | -0.0980* |
|  | (0.0762) | (0.0332) | (0.0531) |
| Constant | 1.179*** | 0.725*** | 0.977*** |
|  | (0.0404) | (0.0495) | (0.0414) |
|  |  |  |  |
| Observations | 6,501 | 5,424 | 7,349 |
| R-squared | 0.025 | 0.032 | 0.032 |
| Number of canton_id | 26 | 26 | 26 |
| Year FE | YES | YES | YES |
| Canton FE | YES | YES | YES |
| Week FE | YES | YES | YES |
| Mean | 1.355 | 0.992 | 1.064 |

Note: Dependent variable defined as Equation (S2). For more details see Table S11.

# Table S14: Staggered Difference-in-Difference: age 60-90

|  | (1) | (2) | (3) |
| --- | --- | --- | --- |
| VARIABLES | Male (60-90) | Female (60-90) | Total (60-90) |
|  |  |  |  |
| Mask | 0.0397** | 0.0423* | 0.0372** |
|  | (0.0169) | (0.0223) | (0.0143) |
| Constant | 3.984*** | 3.744*** | 3.866*** |
|  | (0.0184) | (0.0254) | (0.0153) |
|  |  |  |  |
| Observations | 9,004 | 8,928 | 9,253 |
| R-squared | 0.093 | 0.105 | 0.158 |
| Number of canton_id | 26 | 26 | 26 |
| Year FE | YES | YES | YES |
| Canton FE | YES | YES | YES |
| Week FE | YES | YES | YES |
| Mean | 3.864 | 3.597 | 3.722 |

Note: Dependent variable defined as Equation (S2). For more details see Table S11.

# Table S15: Staggered Difference-in-Difference: age 90+

|  | (1) | (2) | (3) |
| --- | --- | --- | --- |
| VARIABLES | Male 90+ | Female 90+ | Total 90+ |
|  |  |  |  |
| Mask | -0.00363 | 0.0275 | 0.0138 |
|  | (0.0732) | (0.0387) | (0.0415) |
| Constant | 6.470*** | 6.343*** | 6.383*** |
|  | (0.0326) | (0.0159) | (0.0148) |
|  |  |  |  |
| Observations | 7,514 | 8,401 | 8,788 |
| R-squared | 0.114 | 0.210 | 0.247 |
| Number of canton_id | 26 | 26 | 26 |
| Year FE | YES | YES | YES |
| Canton FE | YES | YES | YES |
| Week FE | YES | YES | YES |
| Mean | 6.353 | 6.084 | 6.121 |

Note: Dependent variable defined as Equation (S2). For more details see Table S11.

# Text S4. Impact of Adding Contact Tracing and Social Distancing to Face-Mask Mandates

To assess the impact of adding contact tracing and/or social distancing rules to the face-mask mandates, we defined four mutually exclusive groups of cantons. Group 0 consists of cantons that enacted neither of the three policies. Group 1 consists of cantons that enacted the face-mask mandate plus contact tracing policies (but not social distancing rules). Group 2 consists of cantons that enacted face-mask mandates and social distancing rules (but not contact tracing policies). Finally, Group 3 consists of cantons that implemented all three policies. Formally, we estimated the following equation:

$Log\left( Y_{iwt} \right)= \alpha_{4}+\sum_{i=1}^{3} \rho_{0i}{Group}_{i}+\rho_{1}{Post}_{wt}+ \sum_{i=1}^{3} \rho_{2i}{Did}_{iwt}+\sigma_{c}+\xi_{w}+\phi_{t}+\varepsilon_{iwt} (8)$

where each *Group_i_* is a binary variable indicating whether a canton is in each of the four groups described above. *Post_wt_* is a binary variable equalling 1 for the periods after the implementation of the policy. The parameters of interest are the *ρ*_2_*_i_* for each of the four groups. *ρ*_2_*_i_* estimates the effect of the policy on group *i* compared to group 0. *Did_iwt_* is the interaction between *Group_i_ and Post_wt_*. Finally, *σ_c_*, *ξ_w_* and *φ_t_* are canton, week, and year fixed-effects.

We found that the combination of face-mask mandates with social distancing rules appeared to be most effective (Table S16). Compared to implementing none of the policies (i.e., neither face-mask mandates, contact tracing, nor social distancing rules), this combination resulted in an estimated decrease in all-cause mortality of 5.1% (95% CI: -7.9% to -2.4%). The point estimate for implementing all three measures is 0.6%, which is lower than the one for face-mask mandates plus social distancing rules only. While the point estimate is itself not statistically significantly different from 0, the difference between these policy “packages” was also not statistically significant. We could postulate that too many policies might lead to confusion and a reduction in compliance with induvial policies, but we do not have strong reasons to believe that implementing all three policies would have a smaller impact than introducing just two. Hence, while there is some indication for the effectiveness of the face-mask mandate and social distancing combination we caution against interpreting this as conclusive evidence.

# Table S16. Policy mix

|  | (1) | (2) | (3) | (4) | (5) | (6) | (7) | (8) | (9) |
| --- | --- | --- | --- | --- | --- | --- | --- | --- | --- |
| VARIABLES | Male | Male | Male | Female | Female | Female | Total | Total | Total |
|  |  |  |  |  |  |  |  |  |  |
| Mask + Tracing | -0.18*** | -0.09*** | -0.09*** | -0.25*** | -0.13*** | -0.13*** | -0.21*** | -0.10*** | -0.10*** |
|  | (0.04) | (0.00) | (0.00) | (0.05) | (0.00) | (0.00) | (0.04) | (0.00) | (0.00) |
| Did(1) | -0.01 | -0.01 | -0.01 | 0.12*** | 0.11*** | 0.11*** | 0.05*** | 0.05*** | 0.05*** |
|  | (0.02) | (0.02) | (0.02) | (0.01) | (0.01) | (0.01) | (0.01) | (0.01) | (0.01) |
| Mask + Distancing | 0.08** | 0.17*** | 0.17*** | 0.09* | 0.21*** | 0.21*** | 0.10** | 0.21*** | 0.21*** |
|  | (0.04) | (0.00) | (0.00) | (0.05) | (0.00) | (0.00) | (0.04) | (0.00) | (0.00) |
| Did(2) | -0.03* | -0.03* | -0.03* | -0.06*** | -0.06*** | -0.06*** | -0.05*** | -0.05*** | -0.05*** |
|  | (0.02) | (0.02) | (0.02) | (0.01) | (0.01) | (0.01) | (0.01) | (0.01) | (0.01) |
| Mask + Trac + Dist | -0.07 | -0.01*** | -0.01*** | -0.05 | 0.07*** | 0.07*** | -0.06 | 0.03*** | 0.03*** |
|  | (0.05) | (0.00) | (0.00) | (0.06) | (0.00) | (0.00) | (0.05) | (0.00) | (0.00) |
| Did(3) | -0.03 | -0.03 | -0.03 | 0.02 | 0.02 | 0.02 | -0.01 | -0.01 | -0.01 |
|  | (0.02) | (0.02) | (0.02) | (0.02) | (0.02) | (0.02) | (0.01) | (0.01) | (0.01) |
| Post | -0.05** | -0.05** | 0.01 | -0.12*** | -0.11*** | -0.02 | -0.08*** | -0.08*** | -0.01 |
|  | (0.02) | (0.02) | (0.03) | (0.01) | (0.01) | (0.02) | (0.01) | (0.01) | (0.02) |
| Constant | 2.71*** | 2.62*** | 2.70*** | 2.74*** | 2.62*** | 2.78*** | 2.73*** | 2.63*** | 2.74*** |
|  | (0.04) | (0.00) | (0.01) | (0.05) | (0.00) | (0.02) | (0.04) | (0.00) | (0.01) |
|  |  |  |  |  |  |  |  |  |  |
| Observations | 9,168 | 9,168 | 9,168 | 9,178 | 9,178 | 9,178 | 9,329 | 9,329 | 9,329 |
| R-squared | 0.04 | 0.19 | 0.29 | 0.04 | 0.22 | 0.37 | 0.05 | 0.29 | 0.46 |
| Year FE | NO | NO | YES | NO | NO | YES | NO | NO | YES |
| Canton FE | NO | YES | YES | NO | YES | YES | NO | YES | YES |
| Week FE | NO | NO | YES | NO | NO | YES | NO | NO | YES |
| Mask | -0.01 | -0.01 | -0.01 | 0.04 | 0.04 | 0.04 | 0.00 | 0.00 | 0.00 |
| p-value | 0.76 | 0.71 | 0.70 | 0.09 | 0.11 | 0.11 | 0.00 | 0.92 | 0.00 |
| Tracing | 0.00 | 0.00 | 0.00 | 0.08 | 0.08 | 0.08 | 0.05 | 0.05 | 0.05 |
| p-value | 0.96 | 0.03 | 0.92 | 0.00 | 0.00 | 0.00 | 0.81 | 0.00 | 0.92 |
| Distancing | -0.03 | -0.03 | -0.03 | -0.10 | -0.09 | -0.09 | -0.05 | -0.05 | -0.05 |
| p-value | 0.03 | 0.92 | 0.03 | 0.00 | 0.00 | 0.00 | 0.00 | 0.00 | 0.00 |

Note: Results of regression based on Equation (8); weighted using population as analytical weights. Dependent variable defined as Equation (S1). (a) Cantons in treatment group 1: FR. Did(1) = Group1 X Post; (b) Cantons in treatment group 2: NA, Did(2) = Group2 X Post; (c) Cantons in treatment group 3: BS GE JU SO VD VS ZH, Did(3) = Group3 X Post. (d) Post is equal to 1 for all cantons after July 7. S.E. clustered at a canton level (*** p<0.01, ** p<0.05, * p<0.1). (e) Point estimate of the Test H_0_: (Did3 - Did2 - Did1) X (-1) = 0. (f) Point estimate of the Test H_0_: (Did3 - Did2) = 0. (g) Point estimate of the Test H_0_: (Did3 – Did1) = 0. P-values in (e)-(g) are the significance levels at which H_0_ is rejected.

# Table S17: Policy Mix regression: age 0-29

|  | (1) | (2) | (3) | (4) | (5) | (6) | (7) | (8) | (9) |
| --- | --- | --- | --- | --- | --- | --- | --- | --- | --- |
| VARIABLES | Male  (0-29) | Male  (0-29) | Male  (0-29) | Female (0-29) | Female (0-29) | Female (0-29) | Total  (0-29) | Total  (0-29) | Total  (0-29) |
|  |  |  |  |  |  |  |  |  |  |
| Mask + Tracing | 0.35** | 0.49*** | 0.48*** | 0.42** | 0.50*** | 0.50*** | 0.24** | 0.40*** | 0.40*** |
|  | (0.14) | (0.00) | (0.00) | (0.18) | (0.00) | (0.00) | (0.11) | (0.00) | (0.00) |
| Did(1) | -0.02 | -0.00 | -0.03 | 0.29*** | 0.38*** | 0.39*** | 0.21*** | 0.26*** | 0.25*** |
|  | (0.05) | (0.04) | (0.05) | (0.10) | (0.04) | (0.08) | (0.05) | (0.03) | (0.04) |
| Mask + Distancing | 0.88*** | 1.02*** | 1.02*** | 1.01*** | 1.09*** | 1.07*** | 0.70*** | 0.87*** | 0.87*** |
|  | (0.14) | (0.00) | (0.01) | (0.18) | (0.00) | (0.01) | (0.11) | (0.00) | (0.00) |
| Did(2) | -0.17*** | -0.16*** | -0.25*** | 0.02 | 0.10** | 0.12 | 0.03 | 0.07* | 0.04 |
|  | (0.05) | (0.04) | (0.06) | (0.10) | (0.04) | (0.08) | (0.05) | (0.03) | (0.04) |
| Mask + Trac + Dist | -0.31 | -0.42*** | -0.42*** | -0.35 | -0.52*** | -0.52*** | -0.18 | -0.17*** | -0.16*** |
|  | (0.20) | (0.01) | (0.01) | (0.25) | (0.00) | (0.00) | (0.15) | (0.00) | (0.00) |
| Did(3) | -0.06 | -0.08 | -0.09 | 0.03 | 0.13 | 0.13 | 0.00 | 0.03 | 0.02 |
|  | (0.15) | (0.15) | (0.15) | (0.13) | (0.08) | (0.09) | (0.06) | (0.05) | (0.05) |
| Post | 0.07 | 0.06 | 0.05 | -0.02 | -0.11** | -0.12 | 0.04 | -0.00 | 0.00 |
|  | (0.05) | (0.04) | (0.04) | (0.10) | (0.04) | (0.07) | (0.05) | (0.03) | (0.04) |
| Constant | 0.41*** | 0.27*** | 0.39*** | 0.25 | 0.17*** | 0.18*** | -0.07 | -0.24*** | -0.14** |
|  | (0.14) | (0.00) | (0.08) | (0.18) | (0.00) | (0.06) | (0.11) | (0.00) | (0.06) |
|  |  |  |  |  |  |  |  |  |  |
| Observations | 2,975 | 2,975 | 2,975 | 1,883 | 1,883 | 1,883 | 3,854 | 3,854 | 3,854 |
| R-squared | 0.10 | 0.47 | 0.49 | 0.13 | 0.59 | 0.60 | 0.05 | 0.33 | 0.35 |
| Year FE | NO | NO | YES | NO | NO | YES | NO | NO | YES |
| Canton FE | NO | YES | YES | NO | YES | YES | NO | YES | YES |
| Week FE | NO | NO | YES | NO | NO | YES | NO | NO | YES |
| Mask | -0.13 | -0.08 | -0.18 | 0.28 | 0.35 | 0.38 | 0.24 | 0.29 | 0.26 |
| p-value | 0.40 | 0.61 | 0.65 | 0.04 | 0.00 | 0.00 | 0.48 | 0.00 | 0.79 |
| Tracing | 0.12 | 0.07 | 0.16 | 0.01 | 0.03 | 0.01 | -0.02 | -0.04 | -0.01 |
| p-value | 0.43 | 0.57 | 0.18 | 0.88 | 0.69 | 0.73 | 0.00 | 0.28 | 0.00 |
| Distancing | -0.04 | -0.08 | -0.07 | -0.26 | -0.24 | -0.26 | -0.21 | -0.22 | -0.22 |
| p-value | 0.79 | 0.60 | 0.15 | 0.00 | 0.00 | 0.00 | 0.00 | 0.00 | 0.00 |

Note: Results of regression based on Equation (8); weighted using population as analytical weights. Dependent variable defined as Equation (S2). (a) Cantons in treatment group 1: FR. Did(1) = Group1 X Post; (b) Cantons in treatment group 2: NA, Did(2) = Group2 X Post; (c) Cantons in treatment group 3: BS GE JU SO VD VS ZH, Did(3) = Group3 X Post. (d) Post is equal to 1 for all cantons after July 7. S.E. clustered at a canton level (*** p<0.01, ** p<0.05, * p<0.1). (e) Point estimate of the Test H_0_: (Did3 - Did2 - Did1) X (-1) = 0. (f) Point estimate of the Test H_0_: (Did3 - Did2) = 0. (g) Point estimate of the Test H_0_: (Did3 – Did1) = 0. P-values in (e)-(g) are the significance levels at which H_0_ is rejected.

# Table S18: Policy Mix regression: age 30-59

|  | (1) | (2) | (3) | (4) | (5) | (6) | (7) | (8) | (9) |
| --- | --- | --- | --- | --- | --- | --- | --- | --- | --- |
| VARIABLES | Male (30-59) | Male (30-59) | Male (30-59) | Female (30-59) | Female (30-59) | Female (30-59) | Total (30-59) | Total (30-59) | Total (30-59) |
|  |  |  |  |  |  |  |  |  |  |
| Mask + Tracing | -0.05 | 0.12*** | 0.12*** | 0.01 | 0.18*** | 0.18*** | -0.12*** | -0.05*** | -0.05*** |
|  | (0.04) | (0.00) | (0.00) | (0.05) | (0.00) | (0.00) | (0.03) | (0.00) | (0.00) |
| Did(1) | 0.24*** | 0.23*** | 0.23*** | 0.27*** | 0.27*** | 0.26*** | 0.26*** | 0.25*** | 0.25*** |
|  | (0.03) | (0.03) | (0.03) | (0.04) | (0.04) | (0.05) | (0.04) | (0.04) | (0.04) |
| Mask + Distancing | 0.27*** | 0.43*** | 0.43*** | 0.51*** | 0.67*** | 0.67*** | 0.13*** | 0.21*** | 0.20*** |
|  | (0.04) | (0.00) | (0.00) | (0.05) | (0.00) | (0.00) | (0.03) | (0.00) | (0.00) |
| Did(2) | 0.21*** | 0.21*** | 0.21*** | 0.05 | 0.04 | 0.05 | 0.08* | 0.07* | 0.07 |
|  | (0.03) | (0.03) | (0.03) | (0.04) | (0.04) | (0.04) | (0.04) | (0.04) | (0.04) |
| Mask + Trac + Dist | -0.08 | 0.02*** | 0.02*** | -0.17* | -0.14*** | -0.13*** | -0.07 | -0.04*** | -0.04*** |
|  | (0.06) | (0.00) | (0.00) | (0.09) | (0.00) | (0.00) | (0.05) | (0.00) | (0.00) |
| Did(3) | -0.04 | -0.05 | -0.05 | 0.03 | 0.02 | 0.02 | -0.06 | -0.06 | -0.06 |
|  | (0.06) | (0.06) | (0.06) | (0.05) | (0.05) | (0.05) | (0.05) | (0.05) | (0.05) |
| Post | -0.11*** | -0.11*** | -0.04 | -0.13*** | -0.12*** | -0.05 | -0.13*** | -0.12*** | -0.04 |
|  | (0.03) | (0.03) | (0.05) | (0.04) | (0.04) | (0.06) | (0.04) | (0.04) | (0.05) |
| Constant | 1.19*** | 1.03*** | 1.05*** | 0.79*** | 0.63*** | 0.63*** | 0.96*** | 0.88*** | 0.93*** |
|  | (0.04) | (0.00) | (0.04) | (0.05) | (0.00) | (0.05) | (0.03) | (0.00) | (0.04) |
|  |  |  |  |  |  |  |  |  |  |
| Observations | 6,501 | 6,501 | 6,501 | 5,424 | 5,424 | 5,424 | 7,349 | 7,349 | 7,349 |
| R-squared | 0.01 | 0.14 | 0.16 | 0.04 | 0.22 | 0.24 | 0.01 | 0.11 | 0.13 |
| Year FE | NO | NO | YES | NO | NO | YES | NO | NO | YES |
| Canton FE | NO | YES | YES | NO | YES | YES | NO | YES | YES |
| Week FE | NO | NO | YES | NO | NO | YES | NO | NO | YES |
| Mask | 0.50 | 0.49 | 0.48 | 0.29 | 0.28 | 0.29 | 0.39 | 0.39 | 0.38 |
| p-value | 0.00 | 0.00 | 0.00 | 0.00 | 0.00 | 0.00 | 0.00 | 0.00 | 0.00 |
| Tracing | -0.26 | -0.26 | -0.26 | -0.02 | -0.02 | -0.03 | -0.14 | -0.14 | -0.14 |
| p-value | 0.00 | 0.00 | 0.00 | 0.32 | 0.26 | 0.14 | 0.00 | 0.00 | 0.00 |
| Distancing | -0.28 | -0.28 | -0.28 | -0.24 | -0.24 | -0.24 | -0.31 | -0.31 | -0.31 |
| p-value | 0.00 | 0.00 | 0.00 | 0.00 | 0.00 | 0.00 | 0.00 | 0.00 | 0.00 |

Note: Results of regression based on Equation (8); weighted using population as analytical weights. Dependent variable defined as Equation (S2). (a) Cantons in treatment group 1: FR. Did(1) = Group1 X Post; (b) Cantons in treatment group 2: NA, Did(2) = Group2 X Post; (c) Cantons in treatment group 3: BS GE JU SO VD VS ZH, Did(3) = Group3 X Post. (d) Post is equal to 1 for all cantons after July 7. S.E. clustered at a canton level (*** p<0.01, ** p<0.05, * p<0.1). (e) Point estimate of the Test H_0_: (Did3 - Did2 - Did1) X (-1) = 0. (f) Point estimate of the Test H_0_: (Did3 - Did2) = 0. (g) Point estimate of the Test H_0_: (Did3 – Did1) = 0. P-values in (e)-(g) are the significance levels at which H_0_ is rejected.

# Table S19: Policy Mix regression: age 60-90

|  | (1) | (2) | (3) | (4) | (5) | (6) | (7) | (8) | (9) |
| --- | --- | --- | --- | --- | --- | --- | --- | --- | --- |
| VARIABLES | Male (60-90) | Male (60-90) | Male (60-90) | Female (60-90) | Female (60-90) | Female (60-90) | Total (60-90) | Total (60-90) | Total (60-90) |
|  |  |  |  |  |  |  |  |  |  |
| Mask + Tracing | -0.02 | 0.00*** | 0.00*** | -0.06*** | -0.04*** | -0.04*** | -0.02 | -0.00*** | -0.00*** |
|  | (0.02) | (0.00) | (0.00) | (0.02) | (0.00) | (0.00) | (0.02) | (0.00) | (0.00) |
| Did(1) | -0.05** | -0.05** | -0.05** | 0.04* | 0.04* | 0.04* | -0.01 | -0.01 | -0.01 |
|  | (0.02) | (0.02) | (0.02) | (0.02) | (0.02) | (0.02) | (0.02) | (0.02) | (0.02) |
| Mask + Distancing | 0.12*** | 0.14*** | 0.14*** | 0.05** | 0.06*** | 0.06*** | 0.10*** | 0.11*** | 0.11*** |
|  | (0.02) | (0.00) | (0.00) | (0.02) | (0.00) | (0.00) | (0.02) | (0.00) | (0.00) |
| Did(2) | 0.06** | 0.06** | 0.06** | 0.10*** | 0.09*** | 0.10*** | 0.07*** | 0.07*** | 0.07*** |
|  | (0.02) | (0.02) | (0.02) | (0.02) | (0.02) | (0.02) | (0.02) | (0.02) | (0.02) |
| Mask + Trac + Dist | 0.03 | 0.04*** | 0.03*** | 0.01 | 0.05*** | 0.05*** | 0.02 | 0.03*** | 0.03*** |
|  | (0.02) | (0.00) | (0.00) | (0.03) | (0.00) | (0.00) | (0.02) | (0.00) | (0.00) |
| Did(3) | -0.00 | -0.00 | -0.00 | 0.06** | 0.06** | 0.06** | 0.03 | 0.03 | 0.03 |
|  | (0.03) | (0.03) | (0.03) | (0.03) | (0.03) | (0.03) | (0.02) | (0.02) | (0.02) |
| Post | -0.11*** | -0.11*** | -0.00 | -0.16*** | -0.16*** | -0.04* | -0.13*** | -0.13*** | -0.02 |
|  | (0.02) | (0.02) | (0.03) | (0.02) | (0.02) | (0.02) | (0.02) | (0.02) | (0.02) |
| Constant | 3.86*** | 3.84*** | 3.95*** | 3.59*** | 3.57*** | 3.73*** | 3.73*** | 3.72*** | 3.84*** |
|  | (0.02) | (0.00) | (0.02) | (0.02) | (0.00) | (0.03) | (0.02) | (0.00) | (0.02) |
|  |  |  |  |  |  |  |  |  |  |
| Observations | 9,004 | 9,004 | 9,004 | 8,928 | 8,928 | 8,928 | 9,253 | 9,253 | 9,253 |
| R-squared | 0.01 | 0.05 | 0.14 | 0.01 | 0.06 | 0.15 | 0.02 | 0.08 | 0.21 |
| Year FE | NO | NO | YES | NO | NO | YES | NO | NO | YES |
| Canton FE | NO | YES | YES | NO | YES | YES | NO | YES | YES |
| Week FE | NO | NO | YES | NO | NO | YES | NO | NO | YES |
| Mask | 0.01 | 0.01 | 0.01 | 0.07 | 0.07 | 0.07 | 0.03 | 0.03 | 0.03 |
| p-value | 0.60 | 0.62 | 0.00 | 0.15 | 0.14 | 0.02 | 0.07 | 0.08 | 0.08 |
| Tracing | -0.06 | -0.06 | -0.06 | -0.03 | -0.03 | -0.03 | -0.05 | -0.05 | -0.05 |
| p-value | 0.00 | 0.00 | 0.62 | 0.02 | 0.02 | 0.13 | 0.00 | 0.00 | 0.00 |
| Distancing | 0.04 | 0.04 | 0.05 | 0.03 | 0.03 | 0.03 | 0.04 | 0.04 | 0.04 |
| p-value | 0.00 | 0.00 | 0.00 | 0.08 | 0.09 | 0.08 | 0.00 | 0.00 | 0.00 |

Note: Results of regression based on Equation (8); weighted using population as analytical weights. Dependent variable defined as Equation (S2). (a) Cantons in treatment group 1: FR. Did(1) = Group1 X Post; (b) Cantons in treatment group 2: NA, Did(2) = Group2 X Post; (c) Cantons in treatment group 3: BS GE JU SO VD VS ZH, Did(3) = Group3 X Post. (d) Post is equal to 1 for all cantons after July 7. S.E. clustered at a canton level (*** p<0.01, ** p<0.05, * p<0.1). (e) Point estimate of the Test H_0_: (Did3 - Did2 - Did1) X (-1) = 0. (f) Point estimate of the Test H_0_: (Did3 - Did2) = 0. (g) Point estimate of the Test H_0_: (Did3 – Did1) = 0. P-values in (e)-(g) are the significance levels at which H_0_ is rejected.

# Table S20: Policy Mix regression: age 90+

|  | (1) | (2) | (3) | (4) | (5) | (6) | (7) | (8) | (9) |
| --- | --- | --- | --- | --- | --- | --- | --- | --- | --- |
| VARIABLES | Male 90+ | Male 90+ | Male 90+ | Female 90+ | Female 90+ | Female 90+ | Total 90+ | Total 90+ | Total 90+ |
|  |  |  |  |  |  |  |  |  |  |
| Mask + Tracing | -0.04* | -0.02*** | -0.03*** | -0.09*** | -0.11*** | -0.11*** | -0.05** | -0.09*** | -0.09*** |
|  | (0.02) | (0.00) | (0.00) | (0.02) | (0.00) | (0.00) | (0.02) | (0.00) | (0.00) |
| Did(1) | -0.11** | -0.11** | -0.11** | 0.25*** | 0.25*** | 0.25*** | 0.16*** | 0.15*** | 0.15*** |
|  | (0.05) | (0.05) | (0.05) | (0.02) | (0.02) | (0.02) | (0.02) | (0.02) | (0.02) |
| Mask + Distancing | 0.01 | 0.03*** | 0.03*** | -0.12*** | -0.15*** | -0.15*** | -0.08*** | -0.11*** | -0.11*** |
|  | (0.02) | (0.00) | (0.00) | (0.02) | (0.00) | (0.00) | (0.02) | (0.00) | (0.00) |
| Did(2) | -0.18*** | -0.18*** | -0.18*** | -0.23*** | -0.24*** | -0.24*** | -0.24*** | -0.24*** | -0.24*** |
|  | (0.05) | (0.05) | (0.05) | (0.02) | (0.02) | (0.02) | (0.02) | (0.02) | (0.02) |
| Mask + Trac + Dist | -0.07* | 0.01** | 0.01** | -0.05 | 0.00*** | 0.00*** | -0.05 | -0.01*** | -0.01*** |
|  | (0.04) | (0.00) | (0.00) | (0.04) | (0.00) | (0.00) | (0.04) | (0.00) | (0.00) |
| Did(3) | 0.00 | 0.00 | 0.00 | 0.02 | 0.02 | 0.01 | 0.01 | 0.01 | 0.01 |
|  | (0.06) | (0.06) | (0.06) | (0.03) | (0.03) | (0.03) | (0.03) | (0.03) | (0.03) |
| Post | -0.17*** | -0.17*** | 0.03 | -0.20*** | -0.19*** | 0.00 | -0.18*** | -0.18*** | 0.01 |
|  | (0.05) | (0.05) | (0.06) | (0.02) | (0.02) | (0.02) | (0.02) | (0.02) | (0.03) |
| Constant | 6.26*** | 6.24*** | 6.48*** | 6.06*** | 6.09*** | 6.40*** | 6.13*** | 6.16*** | 6.45*** |
|  | (0.02) | (0.00) | (0.03) | (0.02) | (0.00) | (0.02) | (0.02) | (0.00) | (0.02) |
|  |  |  |  |  |  |  |  |  |  |
| Observations | 7,514 | 7,514 | 7,514 | 8,401 | 8,401 | 8,401 | 8,788 | 8,788 | 8,788 |
| R-squared | 0.01 | 0.08 | 0.18 | 0.02 | 0.07 | 0.25 | 0.02 | 0.06 | 0.28 |
| Year FE | NO | NO | YES | NO | NO | YES | NO | NO | YES |
| Canton FE | NO | YES | YES | NO | YES | YES | NO | YES | YES |
| Week FE | NO | NO | YES | NO | NO | YES | NO | NO | YES |
| Mask | -0.30 | -0.30 | -0.29 | 0.00 | 0.00 | 0.00 | -0.09 | -0.09 | -0.09 |
| p-value | 0.00 | 0.00 | 0.00 | 0.00 | 0.00 | 0.96 | 0.00 | 0.00 | 0.00 |
| Tracing | 0.19 | 0.19 | 0.18 | 0.25 | 0.25 | 0.25 | 0.24 | 0.24 | 0.24 |
| p-value | 0.00 | 0.00 | 0.00 | 0.91 | 0.98 | 0.00 | 0.00 | 0.00 | 0.00 |
| Distancing | 0.11 | 0.11 | 0.11 | -0.24 | -0.24 | -0.24 | -0.15 | -0.15 | -0.15 |
| p-value | 0.00 | 0.00 | 0.01 | 0.00 | 0.00 | 0.00 | 0.00 | 0.00 | 0.00 |

Note: Results of regression based on Equation (8); weighted using population as analytical weights. Dependent variable defined as Equation (S2). (a) Cantons in treatment group 1: FR. Did(1) = Group1 X Post; (b) Cantons in treatment group 2: NA, Did(2) = Group2 X Post; (c) Cantons in treatment group 3: BS GE JU SO VD VS ZH, Did(3) = Group3 X Post. (d) Post is equal to 1 for all cantons after July 7. S.E. clustered at a canton level (*** p<0.01, ** p<0.05, * p<0.1). (e) Point estimate of the Test H_0_: (Did3 - Did2 - Did1) X (-1) = 0. (f) Point estimate of the Test H_0_: (Did3 - Did2) = 0. (g) Point estimate of the Test H_0_: (Did3 – Did1) = 0. P-values in (e)-(g) are the significance levels at which H_0_ is rejected.

# Figure S9: Pre-trend male log all-cause mortality

**
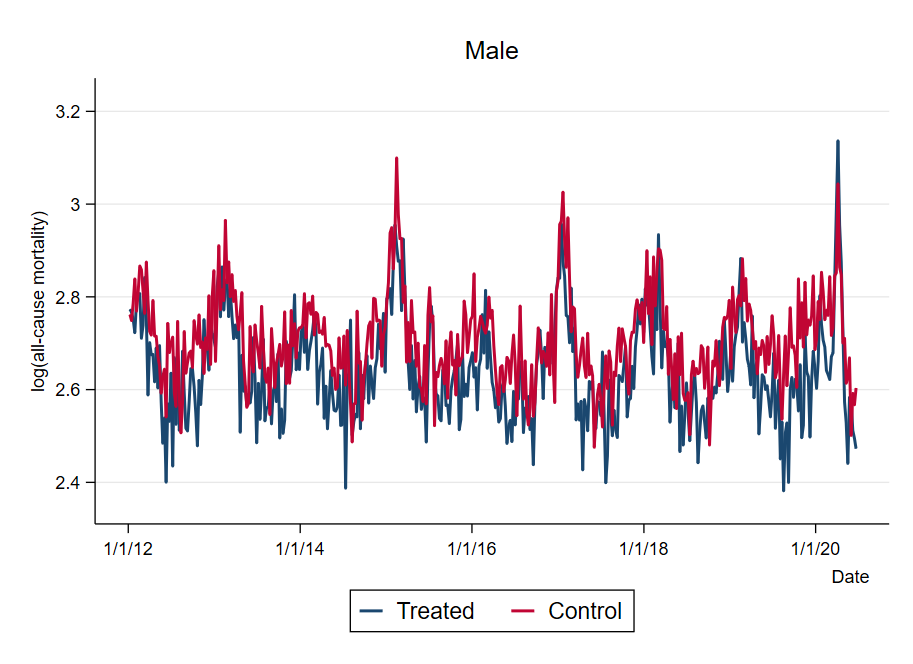
**

Note: Pre-trend period: January 1, 2012 - July 5, 2020. Outcome defined as Equation (S1); weighted using population as analytical weights. Treated cantons (blue line) are those that between July 7 and October 4 have imposed any mask requirement other than Federal indications (e.g., in supermarket, restaurants, open space): BS, FR, GE, JU, NE, SO, VS, VD, ZH. Control cantons (red line) contains the 17 other cantons.

# Figure S10: Pre-trend male log all-cause mortality, LOWESS

**
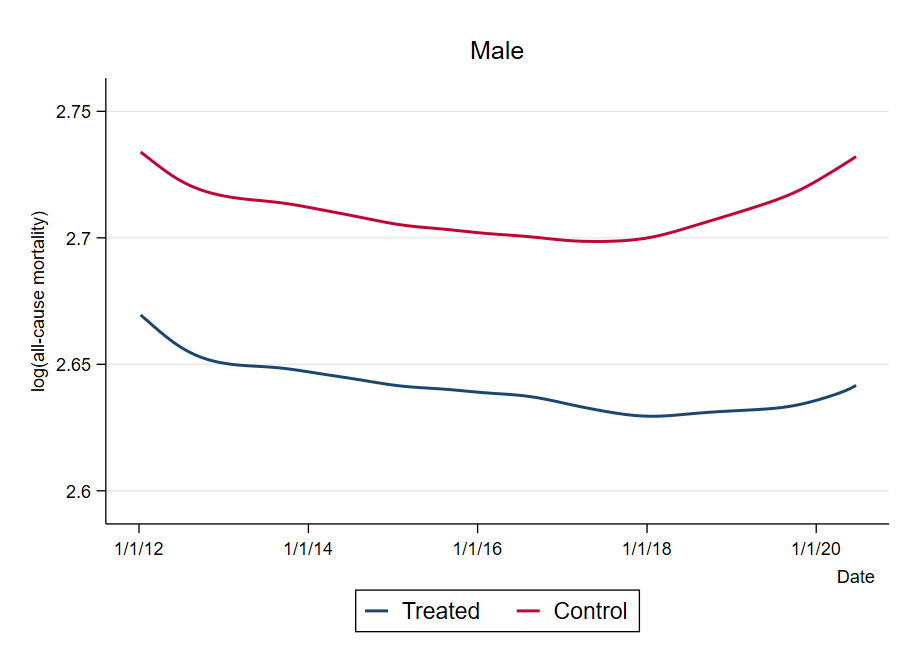
**

Note: Pre-trend period: January 1, 2012 - July 5, 2020. Outcome defined as Equation (S1); weighted using population as analytical weights. Treated cantons (blue line) are those that between July 7 and October 4 have imposed any mask requirement other than Federal indications (e.g., in supermarket, restaurants, open space): BS, FR, GE, JU, NE, SO, VS, VD, ZH. Control cantons (red line) contains the 17 other cantons.

# Figure S11: Pre-trend female log all-cause mortality

**
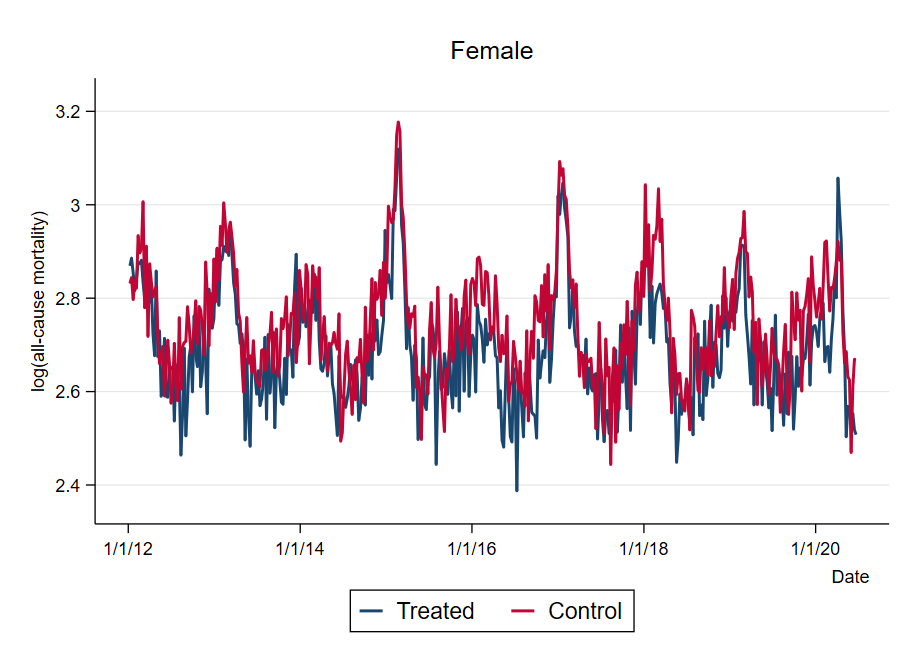
**

Note: Pre-trend period: January 1, 2012 - July 5, 2020. Outcome defined as Equation (S1); weighted using population as analytical weights. Treated cantons (blue line) are those that between July 7 and October 4 have imposed any mask requirement other than Federal indications (e.g., in supermarket, restaurants, open space): BS, FR, GE, JU, NE, SO, VS, VD, ZH. Control cantons (red line) contains the 17 other cantons.

# **Figure S12: Pre-trend male log all-cause mortality, LOWESS**

**
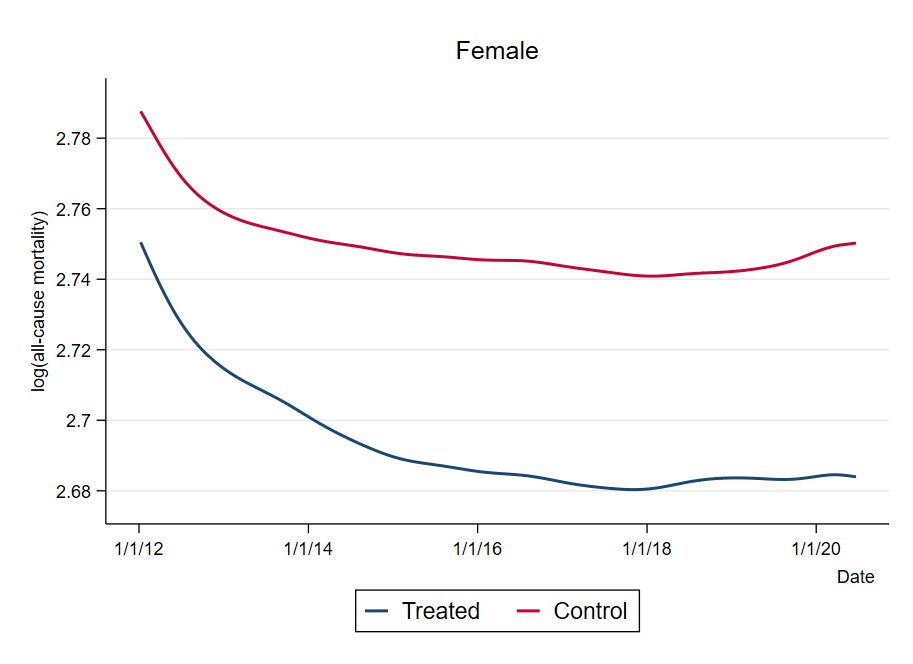
**

Note: Pre-trend period: January 1, 2012 - July 5, 2020. Outcome defined as Equation (S1); weighted using population as analytical weights. Treated cantons (blue line) are those that between July 7 and October 4 have imposed any mask requirement other than Federal indications (e.g., in supermarket, restaurants, open space): BS, FR, GE, JU, NE, SO, VS, VD, ZH. Control cantons (red line) contains the 17 other cantons.

# Figure S13: Pre-trend both sexes log all-cause mortality

**
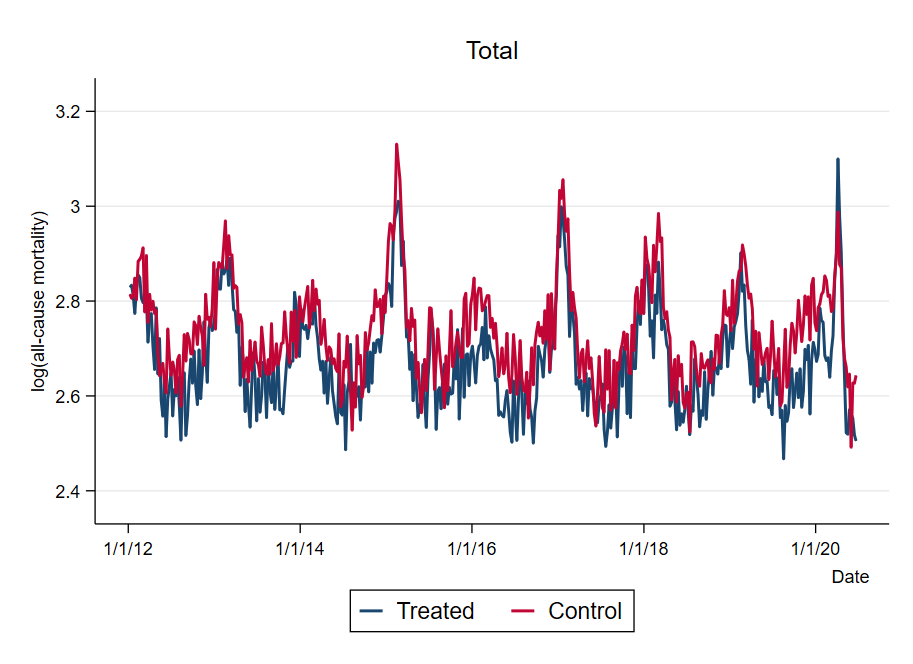
**

Note: Pre-trend period: January 1, 2012 - July 5, 2020. Outcome defined as Equation (S1); weighted using population as analytical weights. Treated cantons (blue line) are those that between July 7 and October 4 have imposed any mask requirement other than Federal indications (e.g., in supermarket, restaurants, open space): BS, FR, GE, JU, NE, SO, VS, VD, ZH. Control cantons (red line) contains the 17 other cantons.

# **Figure S14: Pre-trend both sexes log all-cause mortality, LOWESS**

**
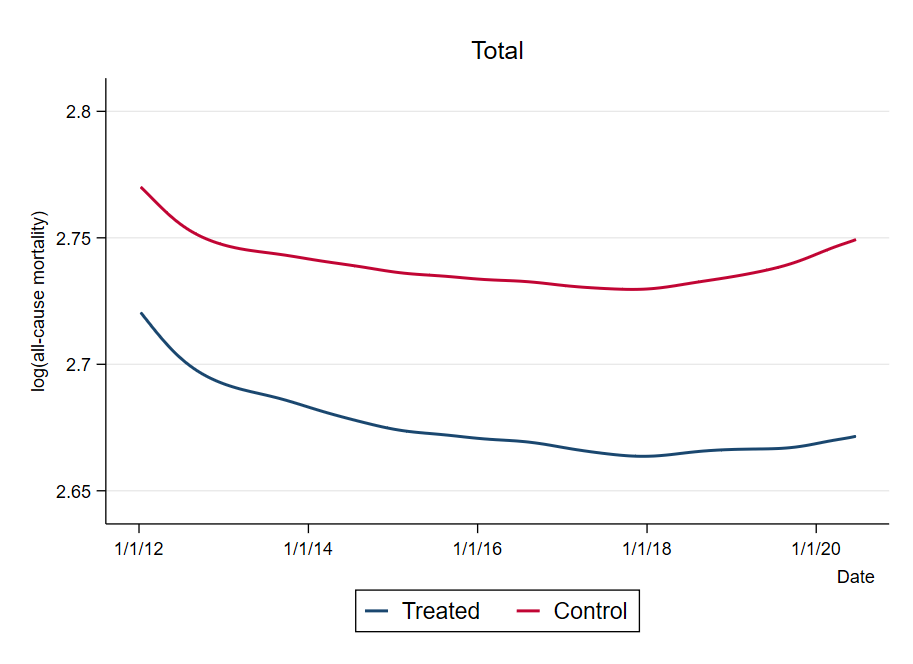
**

Note: Pre-trend period: January 1, 2012 - July 5, 2020. Outcome defined as Equation (S1); weighted using population as analytical weights. Treated cantons (blue line) are those that between July 7 and October 4 have imposed any mask requirement other than Federal indications (e.g., in supermarket, restaurants, open space): BS, FR, GE, JU, NE, SO, VS, VD, ZH. Control cantons (red line) contains the 17 other cantons.

# Text S5: Analysis of covid outcomes

Here we report the estimates of the Difference-in-Difference (Equation (1)) on two covid deaths and cases (Table S22). We narrow the period of analysis to start in March 2020 and add fixed effect accordingly. Figures (7)-(8) illustrate the pre-trends. For covid variables we do not have the demographic characteristic per canton of deaths. Thus, the outcomes in this section are defined as follow:

$CovidCases_{cw}=log\left( \left( \frac{\# of Covid cases_{cw}}{Population_{c,2019}}*100,000 \right)+\sqrt{\left( \left( \frac{\# of Covid cases_{cw}}{Population_{c,2019}}*100,000 \right)^{2}+1 \right)} \right)$ (Equation S4)

$CovidDeaths_{cw}=log\left( \left( \frac{\# of Covid {deaths}_{cw}}{Population_{c,2019}}*100,000 \right)+\sqrt{\left( \left( \frac{\# of Covid {deaths}_{cw}}{Population_{c,2019}}*100,000 \right)^{2}+1 \right)} \right)$ (Equation S5)

Where, $\# of Covid cases_{cw}$and $\# of Covid deathss_{cw}$ are the weekly cases and the deaths in a specific canton in the first 40 weeks of 2020, respectively. Both variables are weighted with the total population of the canton in the previous year (2019). Table (S21) reports the summary statistics for the two variables.

# Table S21: Descriptive Statistics of Covid-19 variables

|  | Mean | SD | Min | Max |
| --- | --- | --- | --- | --- |
| Aggregated Covid Deaths | 4.643 | 1.503 | 0 | 6.551 |
| Weekly Covid Deaths | 0.998 | 1.312 | -1.444 | 5.124 |
| Share Covid Deaths | 0.387 | 0.708 | -1.725 | 3.867 |
| Aggregated Covid Cases | 7.971 | 1.252 | 1.818 | 9.988 |
| Weekly Covid Cases | 4.569 | 1.676 | 0 | 7.950 |
| Share Covid Cases | 2.939 | 1.316 | 0 | 6.289 |
| Observations^a^ | 758 |  |  |  |

Note: (a) Observations in the first 40 weeks of year 2020. Summary statistics of covid-related variables for whole Switzerland. Each row contains the mean of the variable at an aggregate age-class level. Aggregated Covid Deaths and Aggregated Covid Case are the cumulative number in each week. Share Deaths and Share Cases are the log of the IHS transformed ratio between covid deaths/cases in *y* and population divided by 100,000 in the same year *y*.

# Figure S15: Pre-trends of Covid cases share


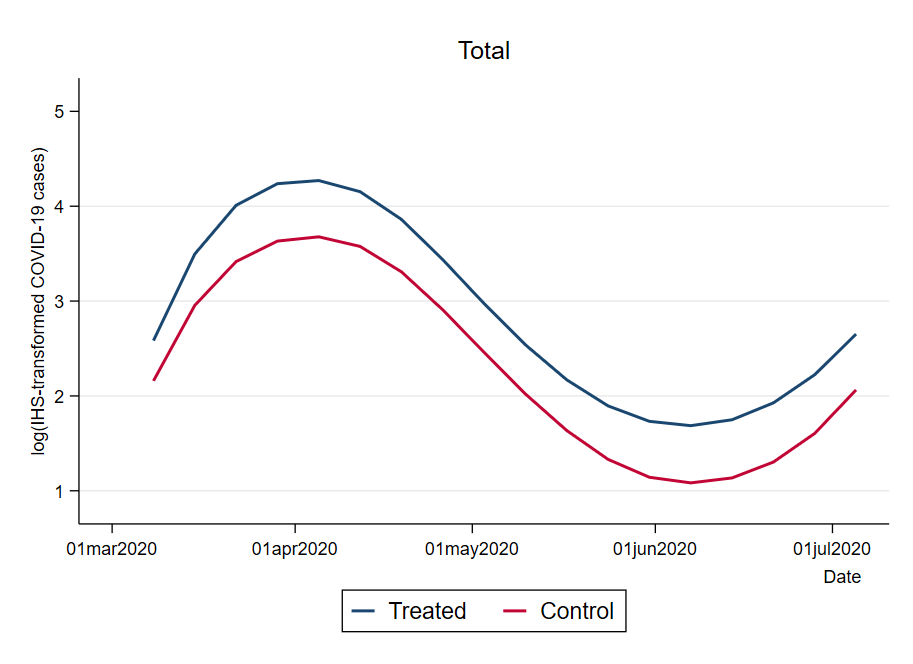


Notes: Pre-trend period: March 2020 - July 5, 2020. Outcome defined as Equation (S4); weighted using population as analytical weights. *Treated* cantons (blue line) are those that between July 7 and October 4 have imposed any mask requirement other than Federal indications (e.g in supermarket, restaurants, open space): BS, FR, GE, JU, NE, SO, VS, VD, ZH. *Control* cantons (red line) contains the 17 other cantons.

# Figure S16: Pre-trends of Covid deaths share


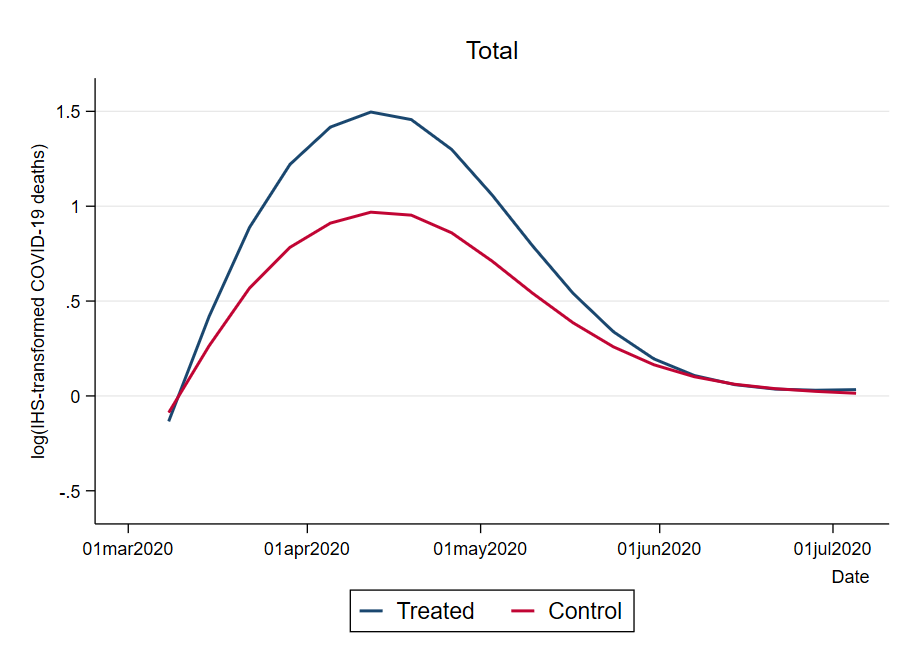


Notes: Pre-trend period: March 2020 - July 5, 2020. Outcome defined as Equation (S5); weighted using population as analytical weights. *Treated* cantons (blue line) are those that between July 7 and October 4 have imposed any mask requirement other than Federal indications (e.g in supermarket, restaurants, open space): BS, FR, GE, JU, NE, SO, VS, VD, ZH. *Control* cantons (red line) contains the 17 other cantons.

# Table S22: Difference-in-Difference regression on covid outcomes

|  | (1) | (2) | (3) | (4) | (5) | (6) |
| --- | --- | --- | --- | --- | --- | --- |
| VARIABLES | Covid deaths | Covid deaths | Covid deaths | Covid cases | Covid cases | Covid cases |
|  |  |  |  |  |  |  |
| Treat | 0.232 | 0.0939 | 0.0949 | 0.583*** | 0.139* | 0.139 |
|  | (0.180) | (0.0717) | (0.0721) | (0.190) | (0.0795) | (0.0813) |
| Post | -0.428*** | -0.427*** | 0.181 | 0.660*** | 0.664*** | 2.225*** |
|  | (0.106) | (0.107) | (0.110) | (0.135) | (0.137) | (0.268) |
| DiD | -0.150 | -0.153 | -0.155 | 0.179 | 0.173 | 0.173 |
|  | (0.169) | (0.171) | (0.172) | (0.187) | (0.190) | (0.194) |
| Constant | 0.472*** | 0.412*** | -0.167** | 2.335*** | 2.560*** | 1.780*** |
|  | (0.101) | (0.0448) | (0.0808) | (0.0915) | (0.0573) | (0.189) |
|  |  |  |  |  |  |  |
| Observations | 788 | 788 | 788 | 788 | 788 | 788 |
| R-squared | 0.143 | 0.229 | 0.711 | 0.143 | 0.196 | 0.853 |
| Canton FE | NO | YES | YES | NO | YES | YES |
| Week FE | NO | NO | YES | NO | NO | YES |

Note: The equation estimated is the same utilized in the regression of the main outcome of interest; weighted using population as analytical weights. *Treat* is the dummy variable for the treatment. Treated cantons are those that between July 7 and October 4th have imposed any mask requirement other than Federal indication (in supermarket, restaurants, open space). Cantons treated: BS, FR, GE, JU, NE, SO, VS, VD, ZH. *Post* is the dummy equal to 1 in the post-policy period. *DiD* is the diff-in-diff coefficient. S.E. clustered at a canton level (*** p<0.01, ** p<0.05, * p<0.1).

# Table S23: Main Difference in differences table – unweighted

|  | (1) | (2) | (3) | (4) | (5) | (6) | (7) | (8) | (9) |
| --- | --- | --- | --- | --- | --- | --- | --- | --- | --- |
| VARIABLES | Male | Male | Male | Female | Female | Female | Total | Total | Total |
|  |  |  |  |  |  |  |  |  |  |
| Treat | 0.007 | -0.009*** | -0.009*** | 0.019 | 0.066*** | 0.066*** | 0.029 | 0.027*** | 0.027*** |
|  | (0.053) | (0.001) | (0.001) | (0.066) | (0.002) | (0.002) | (0.059) | (0.001) | (0.001) |
| Post | -0.045* | -0.048* | -0.005 | -0.124*** | -0.120*** | -0.031 | -0.081*** | -0.081*** | -0.004 |
|  | (0.025) | (0.024) | (0.025) | (0.032) | (0.032) | (0.040) | (0.018) | (0.018) | (0.021) |
| DiD | -0.042 | -0.039 | -0.040 | 0.045 | 0.041 | 0.040 | -0.001 | -0.001 | -0.001 |
|  | (0.032) | (0.031) | (0.031) | (0.044) | (0.044) | (0.044) | (0.022) | (0.022) | (0.023) |
| Constant | 2.686*** | 2.617*** | 2.674*** | 2.716*** | 2.622*** | 2.768*** | 2.700*** | 2.625*** | 2.730*** |
|  | (0.032) | (0.001) | (0.021) | (0.035) | (0.001) | (0.032) | (0.031) | (0.001) | (0.019) |
|  |  |  |  |  |  |  |  |  |  |
| Observations | 9,168 | 9,168 | 9,168 | 9,178 | 9,178 | 9,178 | 9,329 | 9,329 | 9,329 |
| R-squared | 0.001 | 0.116 | 0.166 | 0.003 | 0.152 | 0.229 | 0.004 | 0.171 | 0.263 |
| Year FE | NO | NO | YES | NO | NO | YES | NO | NO | YES |
| Canton FE | NO | YES | YES | NO | YES | YES | NO | YES | YES |
| Week FE | NO | NO | YES | NO | NO | YES | NO | NO | YES |
| Mean | 2.686 | 2.686 | 2.686 | 2.719 | 2.719 | 2.719 | 2.708 | 2.708 | 2.708 |

Note: Results of regression in Equation (1). Column 1-2-3 contain observation for male population. Columns 4-5-6 contain observations for female population. Column 7-8-9 contain observations for aggregate male and female population. S.E. clustered at a canton level (*** p<0.01, ** p<0.05, * p<0.1). Period of estimation: between January 2012 and October 4, 2020. (a) *Treated* cantons are those that between July 7 and October 4 have imposed any mask requirement other than Federal indications (e.g in supermarket, restaurants, open space): BS, FR, GE, JU, NE, SO, VS, VD, ZH. *Post* is equal to 1 for all cantons after July 7.

# Figure S17: Event study – unweighted


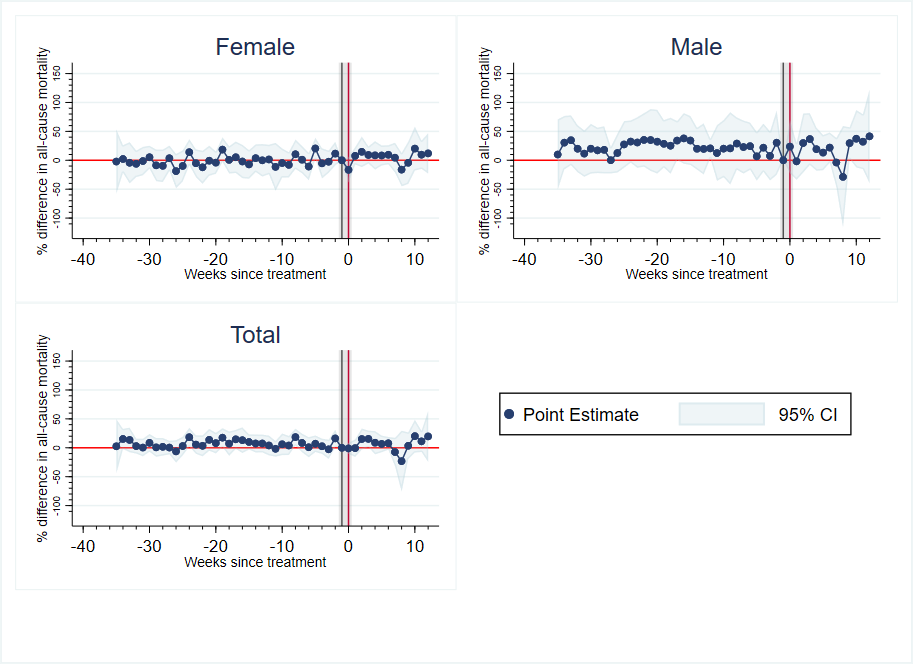


Notes: Estimates of Equation (2). Point estimates (blue points) are displayed along with their 95% confidence intervals (light blue area). The % difference in all-cause mortality is approximated by the log. Baseline period: 1 week prior to the adoption of the face masks policy in each adopting canton, indicated by the solid vertical line in the plot.

# Figure S18: Difference-in-Difference regression with dynamic beta – unweighted


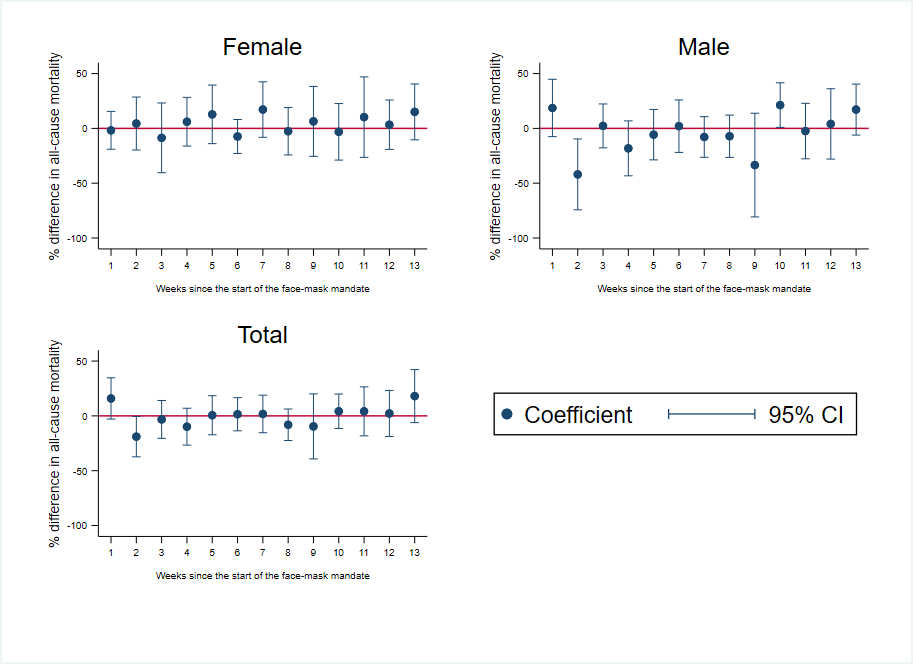


Note: Blue dots are the estimated β_2_ in week *w* as in Equation (7). The % difference in all-cause mortality is approximated by the log. Week 1 is the first week after the treatment, until the 13th week. Outcome defined as Equation (S1). Each bar represents the respective 95% confidence interval.

# **Figure S19: Difference-in-Difference regression on total all-cause mortality with dynamic beta, by age class – unweighted**


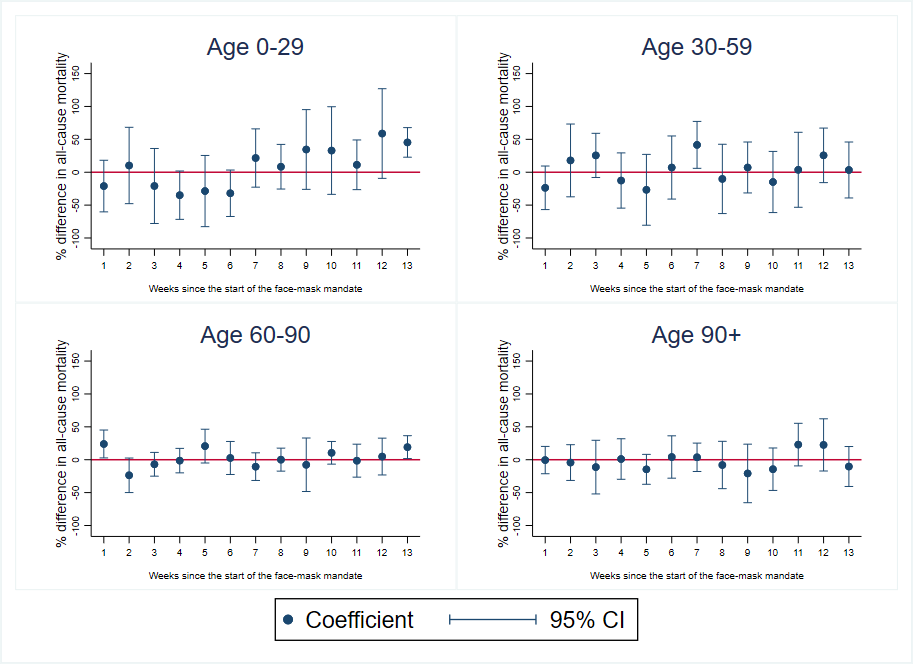


Note: Total population is aggregated male and female. Blue dots are the estimated β_2_ in week *w* as in Equation (7). The % difference in all-cause mortality is approximated by the log. Week 1 is the first week after the treatment, until the 13th week. Outcome defined as Equation (S2). Each bar represents the respective 95% confidence interval.

# Figure S20: Difference-in-Difference regression on covid cases and deaths with dynamic beta – unweighted

**
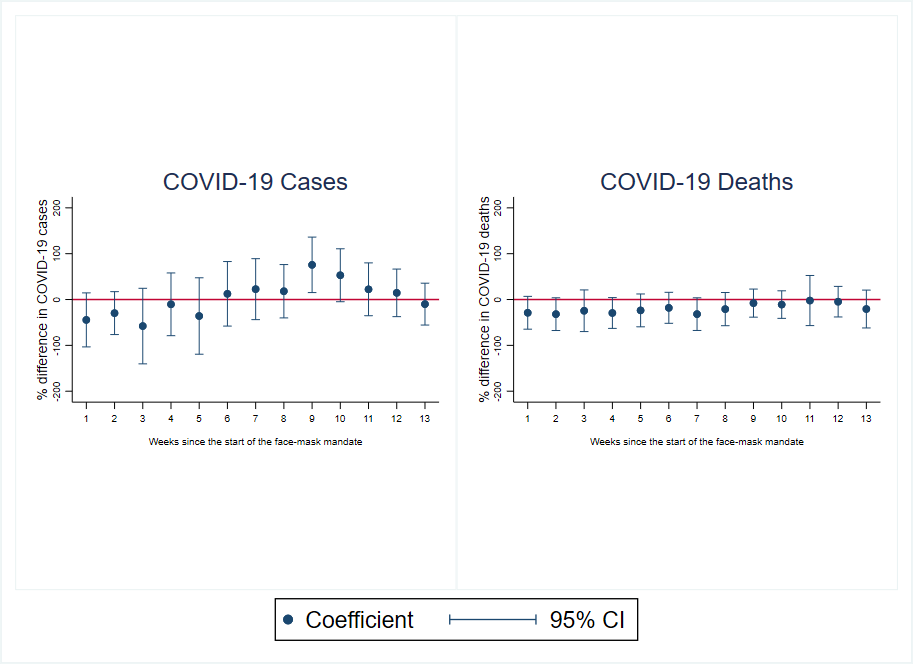
**

Note: Blue dots are the estimated β_2_ in week *w*. The % difference is approximated by the log. Week 1 is the first week after the treatment, until the 13th week. Outcome defined as Equation S4 and S5, respectively. Each bar represents the respective 95% confidence interval.

# Text S6: Treatment effect heterogeneity

The main concern around our estimates regards the staggered implementation of the face-mask policy. In general, with Covid-19-related policy the treatment is usually staggered, and the treatment effect varies over time, thus the two-way fixed-effects estimates might be biased (Goodman-Bacon and Marcus, 2020). The policy we analyze in the present paper has been implemented along different periods between July 7 and October 4. Consider three cantons: Jura, making face-mask mandatory on July 7; Solothurn, making face-mask mandatory on September 3 and Uri, that is never treated. The DiD estimator for Jura will use Uri and Solothurn as control groups; while the DID estimator for Solothurn will use Uri and the already-treated canton (Jura) as control. In general, the bias comes from the fact that the DiD estimates within the control groups use the (early) treatment groups as a control (Goodman-Bacon, 2021). In our main identification (Equation S8) we set a unique starting date for the post-period for all cantons on July 7. This allows us to get rid of likely anticipation bias. In this paragraph we test whether this specification is robust to likely heterogeneous treatment effects. Goodman-Bacon (2021) shows that the two-way fixed-effect DID estimates a variance-weighted average of treatment effect parameters with “positive and negative weights''. When treatment effects do not change overtime, the TWFE DiD yields a variance-weighted average of cross-group treatment effects and all weights are positive. Thus, we follow De Chaisemartin and d’Haultfoeuill (2020) to estimate the weights in our regression model (Equation S8). We found that none of the weight attached to our design is negative. Thus, our TWFE model appears robust to such issues.

# Appendix References

[1] Andrew Goodman-Bacon, and Jan Marcus. Using difference-in-differences to identify causal effects of COVID-19 policies. 2020.

[2] Andrew Goodman-Bacon. Difference-in-differences with variation in treatment timing. *Journal of Econometrics*. 2021.

[3] Clément De Chaisemartin, and Xavier d'Haultfoeuille. Two-way fixed effects estimators with heterogeneous treatment effects. American Economic Review, 110(9):2964-96, 2020.

1. During this period, people were allowed to leave their houses and meet in groups of maximum 5 people, while all bars, clubs, shops and restaurants had to close. Only essential shops (e.g. food and beverages) and health facilities remained opened. In addition, borders with France, Austria and Germany were partly closed. These were re-opened between June 15 and June 22. [↑](#footnote-ref-1)
2. Starting from August 15 wearing the masks became compulsory on airplanes.

   [https://www.bag.admin.ch/bag/en/home/krankheiten/ausbrueche-epidemien-pandemien/](https://www.bag.admin.ch/bag/en/home/krankheiten/ausbrueche-epidemien-pandemien/aktuelle-ausbrueche-epidemien/novel-cov/massnahmen-des-bundes.html) [↑](#footnote-ref-2)
