# Supplementary figures and images for: The impact of face-mask mandates on all-cause mortality in Switzerland: a quasi-experimental study
Source: Eur J Public Health. 2022 Sep 10;32(5):818–24. doi: 10.1093/eurpub/ckac123 (PMC9527954; doi:10.1093/eurpub/ckac123)

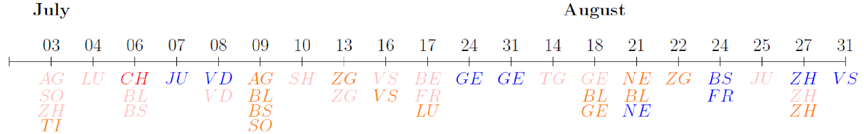

Supplement: ckac123_Supplementary_Data [file ckac123_supplementary_data.zip › ejph-2022-02-om-0080-File006.tif]

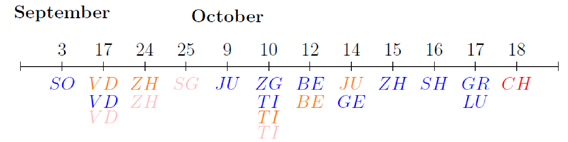

Supplement: ckac123_Supplementary_Data [file ckac123_supplementary_data.zip › ejph-2022-02-om-0080-File007.tif]

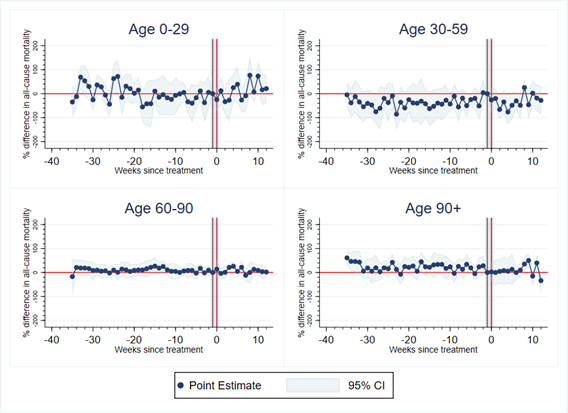

Supplement: ckac123_Supplementary_Data [file ckac123_supplementary_data.zip › ejph-2022-02-om-0080-File008.tif]

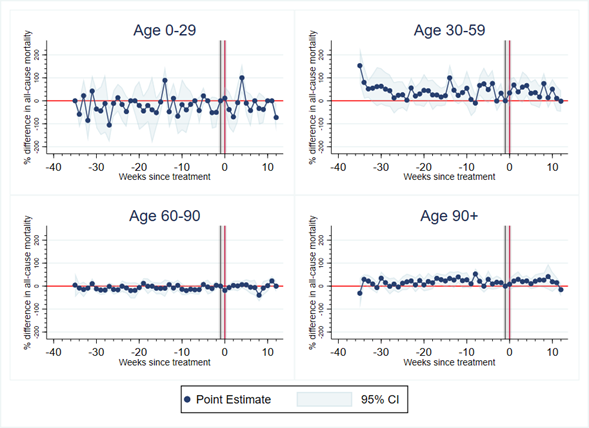

Supplement: ckac123_Supplementary_Data [file ckac123_supplementary_data.zip › ejph-2022-02-om-0080-File009.tif]

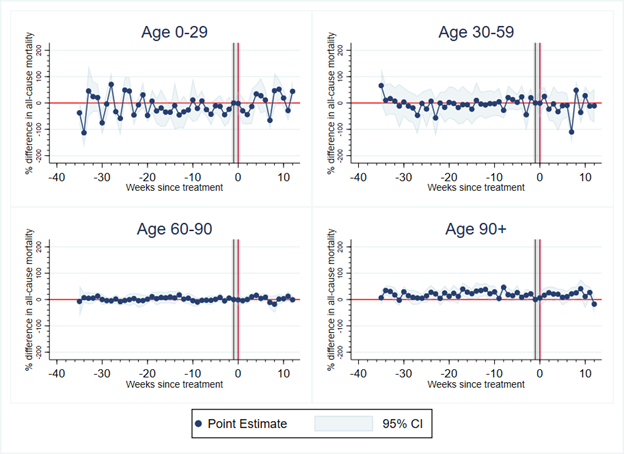

Supplement: ckac123_Supplementary_Data [file ckac123_supplementary_data.zip › ejph-2022-02-om-0080-File010.tif]

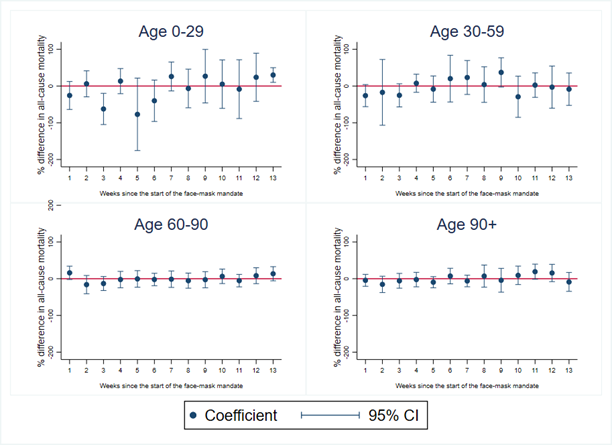

Supplement: ckac123_Supplementary_Data [file ckac123_supplementary_data.zip › ejph-2022-02-om-0080-File011.tif]

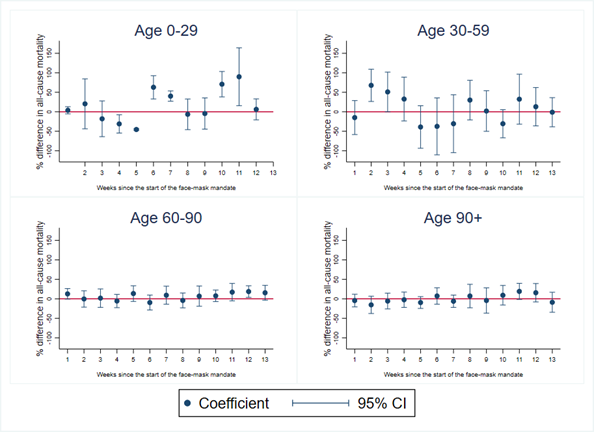

Supplement: ckac123_Supplementary_Data [file ckac123_supplementary_data.zip › ejph-2022-02-om-0080-File012.tif]

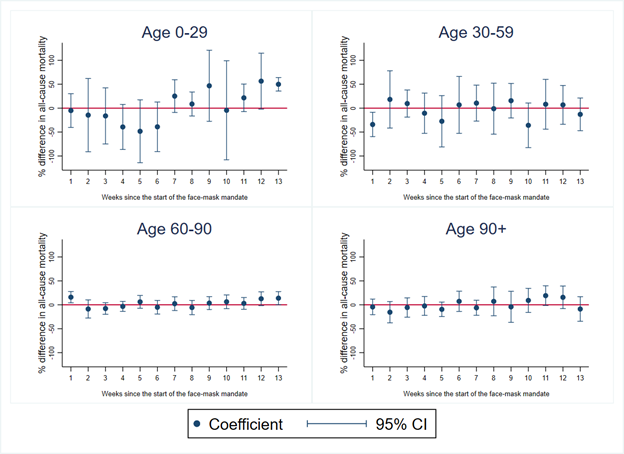

Supplement: ckac123_Supplementary_Data [file ckac123_supplementary_data.zip › ejph-2022-02-om-0080-File013.tif]

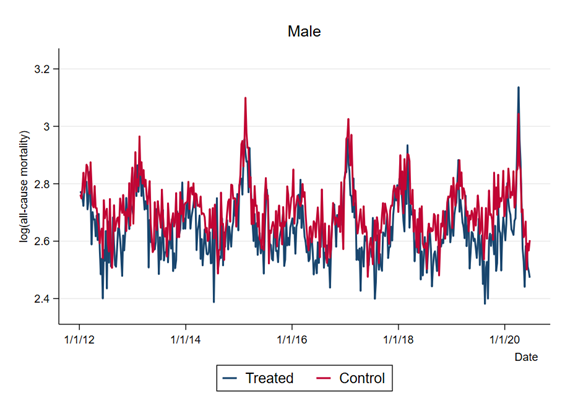

Supplement: ckac123_Supplementary_Data [file ckac123_supplementary_data.zip › ejph-2022-02-om-0080-File014.tif]

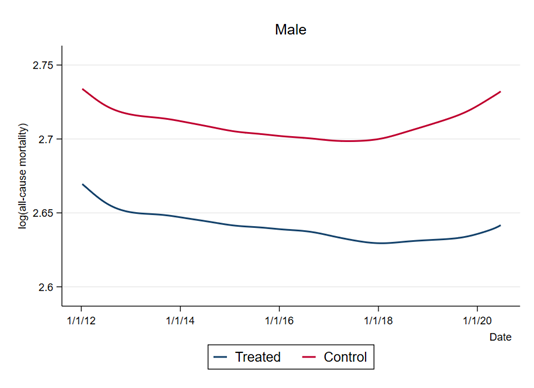

Supplement: ckac123_Supplementary_Data [file ckac123_supplementary_data.zip › ejph-2022-02-om-0080-File015.tif]

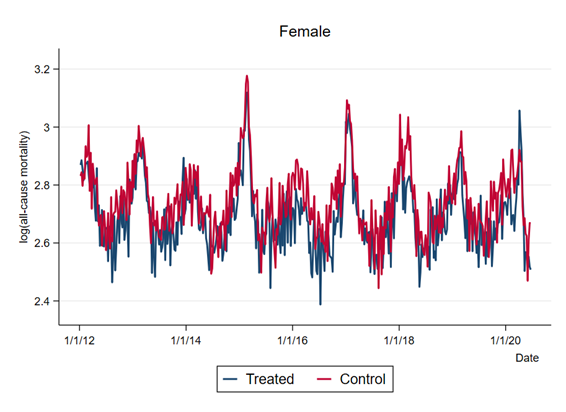

Supplement: ckac123_Supplementary_Data [file ckac123_supplementary_data.zip › ejph-2022-02-om-0080-File016.tif]

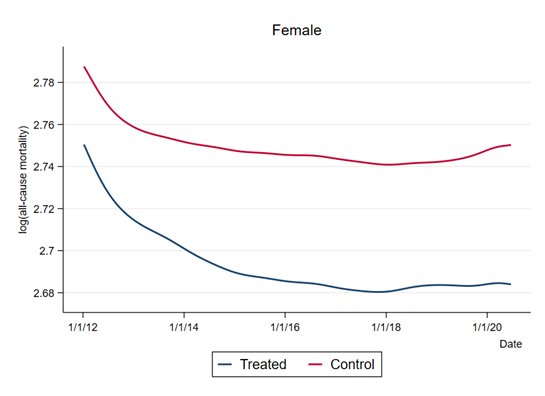

Supplement: ckac123_Supplementary_Data [file ckac123_supplementary_data.zip › ejph-2022-02-om-0080-File017.tif]

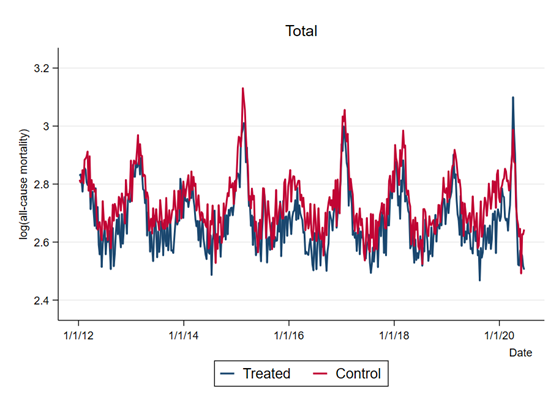

Supplement: ckac123_Supplementary_Data [file ckac123_supplementary_data.zip › ejph-2022-02-om-0080-File018.tif]

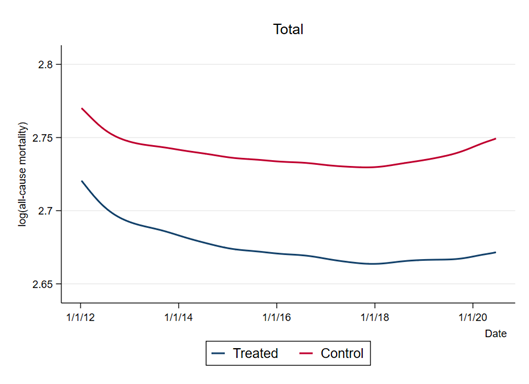

Supplement: ckac123_Supplementary_Data [file ckac123_supplementary_data.zip › ejph-2022-02-om-0080-File019.tif]

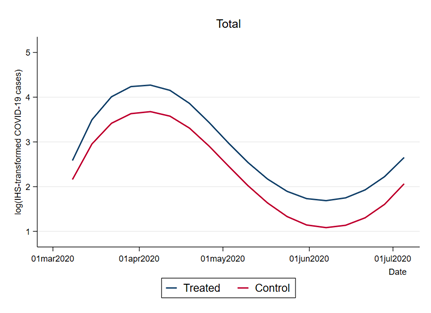

Supplement: ckac123_Supplementary_Data [file ckac123_supplementary_data.zip › ejph-2022-02-om-0080-File020.tif]

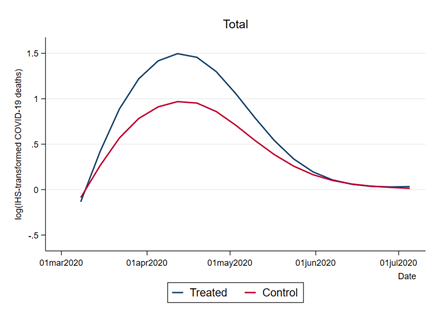

Supplement: ckac123_Supplementary_Data [file ckac123_supplementary_data.zip › ejph-2022-02-om-0080-File021.tif]

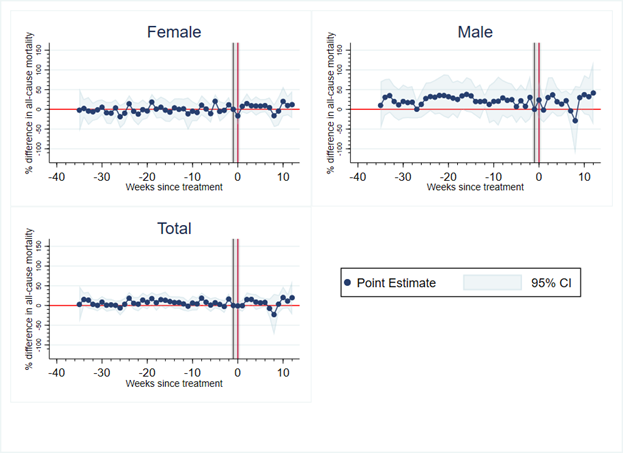

Supplement: ckac123_Supplementary_Data [file ckac123_supplementary_data.zip › ejph-2022-02-om-0080-File022.tif]

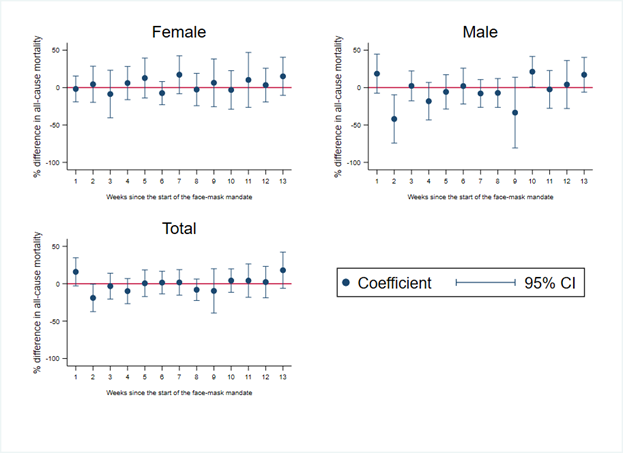

Supplement: ckac123_Supplementary_Data [file ckac123_supplementary_data.zip › ejph-2022-02-om-0080-File023.tif]

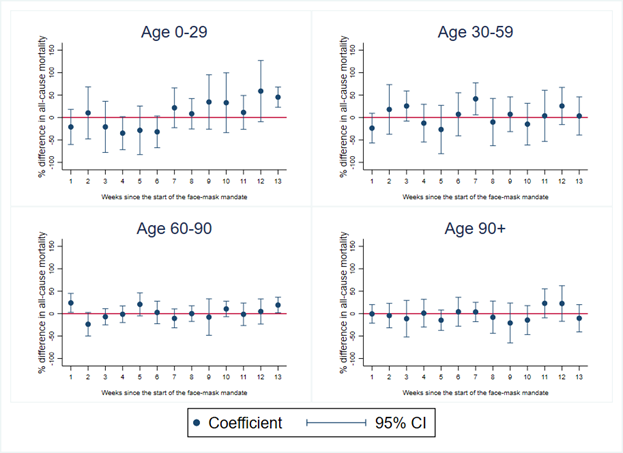

Supplement: ckac123_Supplementary_Data [file ckac123_supplementary_data.zip › ejph-2022-02-om-0080-File024.tif]

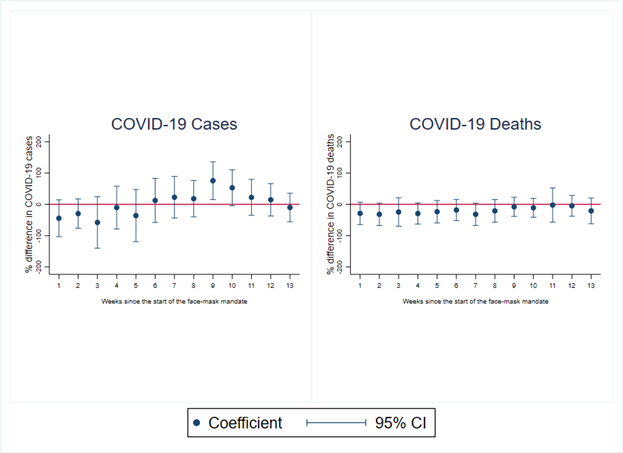

Supplement: ckac123_Supplementary_Data [file ckac123_supplementary_data.zip › ejph-2022-02-om-0080-File025.tif]
